# Supplementary material for: Research on the development trend of global running protection equipment technology from the perspective of patent measurement
Source: PLoS One. 2026 Jan 5;21(1):e0336148. doi: 10.1371/journal.pone.0336148 (PMC12768273; doi:10.1371/journal.pone.0336148)
Supplement: Attachment — Submitted filename: PONE-D-25-26987_reviewer_comments.pdf [file pone.0336148.s001.pdf]

Review Comments for Manuscript ID: PONE-D-25-26987

Title: *Research on the development trend of global running protection equipment technology from the perspective of patent measurement*

General Assessment:

This manuscript presents a comprehensive and methodologically sound patent-based analysis of global technological development in the field of running protective equipment. The study is well-structured, logically organized, and clearly written, making it accessible to a broad audience, including policymakers, industrial stakeholders, and scholars.

The authors have made commendable efforts to categorize and interpret patent data through multiple analytical lenses temporal trends, geographical distributions, key technical domains, and legal status offering valuable insights into the innovation dynamics of the sports protection equipment sector.

Strengths:

1. **Structured Approach and Data Depth**

   The paper distinguishes three distinct phases in the development of patent activity (nascent, accumulation, and rapid growth) and contextualizes this within both global and Chinese patent ecosystems. The segmentation of IPC technologies and focus on key products (running shoes, sportswear, and knee pads) is especially effective in illuminating the state of the art and innovation priorities in this domain (e.g., Figure 3, Table 2).

2. **Relevance for Emerging Economies**

   Although the analysis is partly centered on China's innovation capacity, the framework and methodology presented in this paper could serve as a valuable reference for other nations that are still developing their patent systems or innovation policies. As the authors note, "China entered the field at a relatively late stage, [yet] its innovation capacity and technological influence have shown steady growth" (Conclusion, point 2). This longitudinal view, supported by robust data, offers a replicable model for emerging economies.

3. **Policy and Strategic Implications**

   The manuscript goes beyond descriptive analysis and presents practical policy suggestions (Section: Suggestions), emphasizing IP reform, enterprise innovation, and

industry-academia collaboration. These recommendations make the paper highly relevant for decision-makers and business strategists.

Suggestions for Minor Revisions:

- **Language and Grammar**:
  While the paper is mostly clear, a few minor language improvements could enhance readability. For example, in some sections, overly long sentences could be broken down for better flow. The journal's editorial office may assist in refining this.

- **Clarification on Patent Quality Metrics**:
  The authors provide valuable data on patent validity, noting that over 50% of patents in this domain are classified as invalid primarily due to unpaid maintenance fees or voluntary withdrawal (Figure 4). This indicates a potential issue with the overall quality or commercial viability of many patents in the dataset. While the authors have done a thorough job in collecting comprehensive patent data using multiple keywords and IPC classifications (lines 109–117, section: Analysis of basic patent information), it would strengthen the study if they briefly addressed how this high invalidation rate affects the reliability of using **total patent volume** as a proxy for innovation trends. For instance, are trend analyses based on **all patents**, or do they account for **patent quality**, such as validity, citations, or commercial outcomes? A short reflection on this limitation would enhance the interpretability and robustness of the findings.

Recommendation:

I recommend this manuscript for publication in *PLOS ONE* after minor revisions. It serves as an insightful and practical contribution to the field of sports technology innovation and patent analytics. The methodological approach and findings can benefit not only China but also other countries looking to enhance their intellectual property systems and support high-tech manufacturing in the sports industry.
