# Supplementary material for: Research on the development trend of global running protection equipment technology from the perspective of patent measurement
Source: PLoS One. 2026 Jan 5;21(1):e0336148. doi: 10.1371/journal.pone.0336148 (PMC12768273; doi:10.1371/journal.pone.0336148)
Supplement: Attachment — Submitted filename: PONE-D-25-26987 DARA.pdf [file pone.0336148.s002.pdf]

PLOS ONE

Research on the development trend of global running protection equipment technology from the perspective of patent measurement

--Manuscript Draft--

**Manuscript Number:**	PONE-D-25-26987
**Article Type:**	Research Article
**Full Title:**	Research on the development trend of global running protection equipment technology from the perspective of patent measurement
**Short Title:**	Patent-Based Analysis of Running Protection Technologies
**Corresponding Author:**	Yao Liu, Ph.D<br>Wuxi Taihu University<br>wuxi, jiangsu CHINA
**Keywords:**	Running protective equipment; Patent; Patent measurement; Technology development
**Abstract:**	To provide a comprehensive analysis of global development trends in running protective equipment technology, this study investigates the evolution and technological trajectories of the field by conducting an in-depth examination of domestic and international patent literature, utilizing advanced patent mining and data analysis methodologies. The findings identify three distinct phases in the progression of global patent activity within this domain: the nascent stage (1898–1970), the technological accumulation period (1971–1999), and the rapid advancement phase post-2000. Notably, American enterprises have established and continue to maintain a dominant position in this field. Although China's engagement in research and development (R&D) commenced relatively late, its innovation capacity and global influence have demonstrated steady growth.Invention patents constitute the majority of intellectual property in this sector; however, the economic returns generated by these patents often fail to offset their maintenance costs. This imbalance poses significant challenges to commercialization and limits the economic viability of innovations. Current R&D efforts are primarily focused on running shoes, sportswear, and knee pads. Key technological advancements and patent strategies in this field emphasize areas such as sole stabilization plates, compressible midsole materials, shoe support systems, optimization of textile materials, innovations in garment stitching techniques, integration of protective features into clothing, structural design of knee pads, and improvements in cushioning mechanisms.
**Order of Authors:**	Yao Liu, Ph.D
	Qiong SHEN
	Lei QIANG
**Additional Information:**	
**Question**	**Response**
**Financial Disclosure**<br><br>Enter a financial disclosure statement that describes the sources of funding for the work included in this submission. Review the submission guidelines for detailed requirements. View published research articles from *PLOS ONE* for specific examples.<br><br>This statement is required for submission	Yes

Powered by Editorial Manager® and ProduXion Manager® from Aries Systems Corporation

and **will appear in the published article** if the submission is accepted. Please make sure it is accurate.<br><br>**Funded studies**<br>Enter a statement with the following details:<br>• Initials of the authors who received each award<br>• Grant numbers awarded to each author<br>• The full name of each funder<br>• URL of each funder website<br>• Did the sponsors or funders play any role in the study design, data collection and analysis, decision to publish, or preparation of the manuscript?<br><br>Did you receive funding for this work?	
Please add funding details.<br>as follow-up to "**Financial Disclosure**<br><br>Enter a financial disclosure statement that describes the sources of funding for the work included in this submission. Review the submission guidelines for detailed requirements. View published research articles from *PLOS ONE* for specific examples.<br><br>This statement is required for submission and **will appear in the published article** if the submission is accepted. Please make sure it is accurate.<br><br>**Funded studies**<br>Enter a statement with the following details:<br>• Initials of the authors who received each award<br>• Grant numbers awarded to each author<br>• The full name of each funder<br>• URL of each funder website<br>• Did the sponsors or funders play any role in the study design, data collection and analysis, decision to publish, or preparation of the manuscript?<br><br>Did you receive funding for this work?"	基金编号：19BTY060，项目名称：长三角户外运动产业跨界融合发展研究

Please select the country of your main research funder (please select carefully as in some cases this is used in fee calculation).<br>as follow-up to "**Financial Disclosure**<br><br>Enter a financial disclosure statement that describes the sources of funding for the work included in this submission. Review the submission guidelines for detailed requirements. View published research articles from *PLOS ONE* for specific examples.<br><br>This statement is required for submission and **will appear in the published article** if the submission is accepted. Please make sure it is accurate.<br><br>**Funded studies**<br>Enter a statement with the following details:<br>• Initials of the authors who received each award<br>• Grant numbers awarded to each author<br>• The full name of each funder<br>• URL of each funder website<br>• Did the sponsors or funders play any role in the study design, data collection and analysis, decision to publish, or preparation of the manuscript?<br><br>Did you receive funding for this work?"	CHINA - CN
**Competing Interests**<br><br>Use the instructions below to enter a competing interest statement for this submission. On behalf of all authors, disclose any competing interests that could be perceived to bias this work—acknowledging all financial support and any other relevant financial or non-financial competing interests.<br><br>This statement is required for submission and **will appear in the published article** if the submission is accepted. Please make sure it is accurate and that any funding	作者声明不存在利益冲突。

sources listed in your Funding Information later in the submission form are also declared in your Financial Disclosure statement.<br><br>View published research articles from *PLOS ONE* for specific examples.<br><br>**NO authors have competing interests**<br><br>Enter: *The authors have declared that no competing interests exist.*<br><br>**Authors with competing interests**<br><br>Enter competing interest details beginning with this statement:<br><br>*I have read the journal's policy and the authors of this manuscript have the following competing interests: [insert competing interests here]*<br><br>* typeset	
**Ethics Statement**<br><br>Enter an ethics statement for this submission. This statement is required if the study involved:<br><br>• Human participants<br>• Human specimens or tissue<br>• Vertebrate animals or cephalopods<br>• Vertebrate embryos or tissues<br>• Field research<br><br>Write "N/A" if the submission does not require an ethics statement.<br><br>General guidance is provided below. Consult the submission guidelines for detailed instructions. **Make sure that all information entered here is included in the Methods section of the manuscript.**	N/A

Powered by Editorial Manager® and ProduXion Manager® from Aries Systems Corporation

**Format for specific study types**<br><br>**Human Subject Research (involving human participants and/or tissue)**<br>• Give the name of the institutional review board or ethics committee that approved the study<br>• Include the approval number and/or a statement indicating approval of this research<br>• Indicate the form of consent obtained (written/oral) or the reason that consent was not obtained (e.g. the data were analyzed anonymously)<br><br>**Animal Research (involving vertebrate animals, embryos or tissues)**<br>• Provide the name of the Institutional Animal Care and Use Committee (IACUC) or other relevant ethics board that reviewed the study protocol, and indicate whether they approved this research or granted a formal waiver of ethical approval<br>• Include an approval number if one was obtained<br>• If the study involved *non-human primates*, add *additional details* about animal welfare and steps taken to ameliorate suffering<br>• If anesthesia, euthanasia, or any kind of animal sacrifice is part of the study, include briefly which substances and/or methods were applied<br><br>**Field Research**<br><br>Include the following details if this study involves the collection of plant, animal, or other materials from a natural setting:<br>• Field permit number<br>• Name of the institution or relevant body that granted permission	
**Data Availability**<br><br>Authors are required to make all data underlying the findings described fully available, without restriction, and from the time of publication. PLOS allows rare exceptions to address legal and ethical concerns. See the PLOS Data Policy and FAQ for detailed information.	Yes - all data are fully available without restriction

A Data Availability Statement describing where the data can be found is required at submission. Your answers to this question constitute the Data Availability Statement and **will be published in the article**, if accepted.<br><br>**Important:** Stating 'data available on request from the author' is not sufficient. If your data are only available upon request, select 'No' for the first question and explain your exceptional situation in the text box.<br><br>Do the authors confirm that all data underlying the findings described in their manuscript are fully available without restriction?	
**Describe where the data may be found in full sentences. If you are copying our sample text, replace any instances of XXX with the appropriate details.**<br><br>• If the data are **held or will be held in a public repository**, include URLs, accession numbers or DOIs. If this information will only be available after acceptance, indicate this by ticking the box below. For example: *All XXX files are available from the XXX database (accession number(s) XXX, XXX.).*<br>• If the data are all contained **within the manuscript and/or Supporting Information files**, enter the following: *All relevant data are within the manuscript and its Supporting Information files.*<br>• If neither of these applies but you are able to provide **details of access elsewhere**, with or without limitations, please do so. For example:<br><br>*Data cannot be shared publicly because of [XXX]. Data are available from the XXX Institutional Data Access / Ethics Committee (contact via XXX) for researchers who meet the criteria for access to confidential data.*<br><br>*The data underlying the results presented in the study are available from (include the name of the third party*	所有相关数据均在手稿及其支持信息文件中。

and contact information or URL).<br>• This text is appropriate if the data are owned by a third party and authors do not have permission to share the data.<br><br>* typeset	
Additional data availability information:	Tick here if the URLs/accession numbers/DOIs will be available only after acceptance of the manuscript for publication so that we can ensure their inclusion before publication.

Powered by Editorial Manager® and ProduXion Manager® from Aries Systems Corporation

手稿 Click here to access/download;Manuscript;renamed_f90d3.docx

Research on the development trend of global running protection equipment technology from the perspective of patent measurement

LIU Yao[1*,#a], SHEN Qiong,[2, #a] QIANG Lei[3, #a]

[#a] College of Basic Education, Wuxi Taihu University, Wuxi, China

* Corresponding author: LIU Yao

15061116221@163.com

Conceptualization: SHEN Qiong, LIU Yao, QIANG Lei

Data curation: SHEN Qiong, LIU Yao

Formal analysis: SHEN Qiong, LIU Yao

Investigation: LIU Yao, QIANG Lei

Methodology: SHEN Qiong, LIU Yao

Validation: SHEN Qiong, QIANG Lei

Writing – original draft: SHEN Qiong, LIU Yao

Writing – review & editing:SHEN Qiong, LIU Yao

Abstract

To provide a comprehensive analysis of global development trends in running protective equipment technology, this study investigates the evolution and technological trajectories of the field by conducting an in-depth examination of domestic and international patent literature, utilizing advanced patent mining and data analysis methodologies. The findings identify three distinct phases in the progression of global patent activity within this domain: the nascent stage (1898–1970), the technological accumulation period (1971–1999), and the rapid advancement phase post-2000. Notably, American enterprises have established and continue to maintain a dominant position in this field. Although China's engagement in research and development (R&D) commenced relatively late, its innovation capacity and global influence have demonstrated steady growth.Invention patents constitute the majority of intellectual property in this sector; however, the economic returns generated by these patents often fail to offset their maintenance costs. This imbalance poses significant challenges to commercialization and limits the economic viability of innovations. Current R&D efforts are primarily focused on running shoes, sportswear, and knee pads. Key technological advancements and patent strategies in this field emphasize areas such as sole stabilization plates, compressible midsole materials, shoe support systems, optimization of textile materials, innovations in garment stitching techniques, integration of protective features into clothing, structural design of knee pads, and improvements in cushioning mechanisms.

Keywords

Running protective equipment; Patent; Patent measurement; Technology development

Introduction

Since the issuance of the "Several Opinions on Accelerating the Development of the Sports Industry and Promoting Sports Consumption", China's sports industry has entered a period of rapid growth, beginning in 2014 [1]. This sector has emerged as a significant driver of national economic development. However, with continuous improvements in living standards, consumer preferences have become increasingly diverse and personalized. This shift has exposed a clear supply-demand imbalance in China's sporting goods market, with the current supply of equipment no longer able to meet changing consumer expectations.The report from the 20th National Congress of the Communist Party of China emphasizes that "high-quality development is the foremost task in the comprehensive construction of a modern socialist country" [2]. This directive highlights key deficiencies within China's sports industry, particularly the inadequate capacity for scientific research and technological innovation. These limitations have resulted in many domestic products being relegated to the lower tiers of the global value chain [3]. In this context, the failure to transition from traditional manufacturing models to an innovation-driven development paradigm will severely hamper the industry's ability to align with contemporary societal demands and global trends [4].Promoting high-quality development within the sports sector is therefore essential to addressing the supply-demand gap, enhancing industrial competitiveness, and elevating China's position within the global sports equipment market.

Running is widely regarded as a fundamental activity for promoting national fitness. However, during running, sustained mechanical stress on the limbs, combined with repetitive friction among skeletal muscles and irregular movement patterns, frequently leads to various physical injuries and conditions [5]. These include knee joint degeneration, tibial stress syndrome, plantar fasciitis, muscle spasms, and other related ailments [6]. Empirical evidence indicates that protective equipment represents the most effective strategy for both preventing and managing such issues. This approach is associated with high levels of user satisfaction, strong compliance rates, and significant protective benefits.In 2021, the revenue of running-related products in China has reached 183.12 billion yuan, showing the huge market of the industry. Nevertheless, as the manufacturing of running protective equipment is closely tied to human movement science, it must adhere to rigorous quality and technical standards, necessitating substantial research and development (R&D) efforts to drive innovation. Simultaneously, key technologies in this domain remain concentrated in a small number of countries and are tightly safeguarded as trade secrets by leading enterprises. Consequently, domestic running protective equipment often lacks high value-added core technologies, exhibits suboptimal service performance, and faces limited competitiveness in the consumer market.To address these challenges, it is crucial to analyze global development trends in running protective equipment technology, increase investments in scientific research, and overcome the technical barriers imposed by technologically advanced nations. Advancing the research and development of high-end running protective equipment is vital for fostering the growth of quality-driven sports industries and strengthening the nation’s position in the global market.

Patents, as a critical embodiment of scientific and technological innovation, serve as a reliable indicator of emerging trends in scientific and technological progress [7]. According to estimates by the World Intellectual Property Organization (WIPO), the effective analysis and application of patent information can shorten the average research and development (R&D) cycle of enterprises by 60% and reduce scientific research expenditures by 40%. Consequently, in promoting the high-quality development of the sports industry, it is imperative for relevant authorities to uncover and capitalize on the substantial value embedded within patent data. In recent years, numerous scholars have investigated the technological evolution of sports-related products through patent analysis, providing competitive intelligence to support product R&D in sports manufacturing enterprises. For instance, Wang Jun (2013) conducted a comprehensive study of technological development trends in the football industry using patent data mining [8]. The analysis addressed multiple dimensions, including spatial and temporal distribution, patent types, and technical comparisons. The study identified key technical priorities in the field, such as materials used in football production, manufacturing processes, and auxiliary facilities.Similarly, Ming Yu et al. (2013) utilized patent analysis to conduct a comparative study of advancements in sports footwear technologies between Chinese sports enterprises and Nike [9]. Their findings indicated that Nike's input-output ratio in patent applications significantly exceeded that of Chinese companies, demonstrating superior integration, applicability, and innovation in R&D technologies. Yin Long et al. (2016) examined the development status of golf club technologies from a patent perspective [10]. The authors proposed that Chinese enterprises should increase their R&D investments in golf club technologies to establish competitive advantages for domestic brands. Additionally, Wan Yu et al. (2018) conducted a systematic analysis of the technological development trajectories, R&D priorities, and geographical distribution of international sports equipment patents [11]. Their findings revealed that the primary technological hotspots in sports equipment are concentrated in fitness apparatus, competitive gear, and training devices. Building on these foundational studies, the present research focuses on a targeted patent analysis of running protective equipment to elucidate technical development trends within this domain. This analysis aims to provide theoretical support for decision-making in technology R&D and patent strategies for China's running protective equipment industry. Moreover, it seeks to advance the high-quality development of the nation's broader sports industry.

Analysis of basic patent information

In this study, the terms "Protective running gear" and "Running protective gear" were initially employed to conduct a search in the Patsnap global patent database. To improve the comprehensiveness and accuracy of the retrieved data, additional English keywords, such as "Running shoes," "Knee pads for running," "Leggings for running," "Waist support for running," and "Ankle support," were incorporated based on a review of relevant literature. Additionally, International Patent Classification (IPC) codes were applied to further refine the search strategy. As of January 2025, a total of 4,407 patents had been granted in this field. However, due to a maximum lag time of up to 18 months between patent application and publication, the number of patent applications filed after 2023 may not fully reflect recent trends in technological

development. Consequently, the data presented in this study is intended for illustrative purposes only and should be interpreted with caution.

Analysis of global patent application trends

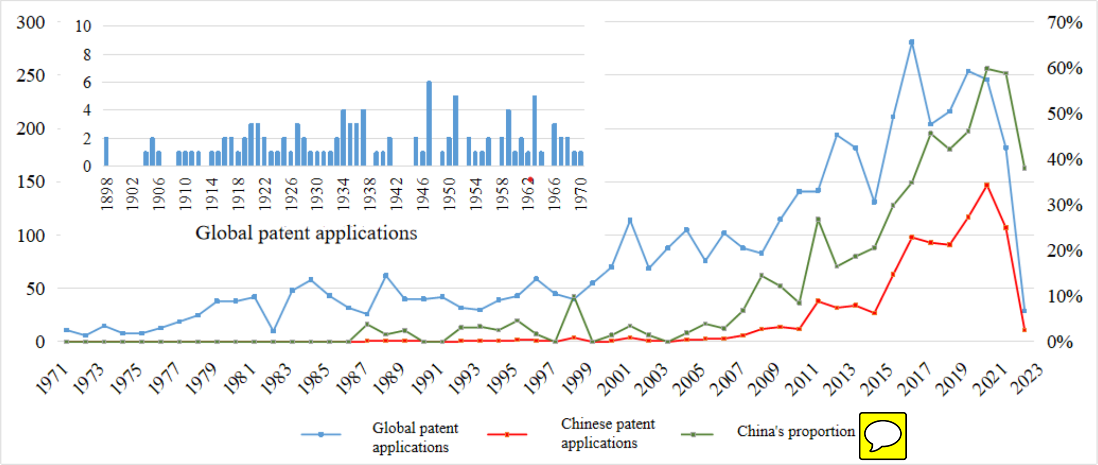

Figure 1 Trend of global patent application in the field of running protective equipment

Figure 1 presents the temporal trends in global and Chinese patent applications within the domain of running protective equipment, as well as the changing proportion of Chinese filings relative to global submissions. The data indicate that patent activity in this field dates back to 1898. From 1898 to 1970, technological advancement in this area was intermittent, with annual patent filings consistently remaining in the single digits. This period is therefore considered the nascent stage of innovation in running protective equipment, primarily marked by the foundational accumulation of technological knowledge.Between 1971 and 1999, rising material living standards and heightened public health consciousness catalyzed the emergence of a nationwide fitness movement. During this period, both official promotion and grassroots initiatives contributed to the widespread popularity of running events such as the New York, Berlin, and Honolulu Marathons. In parallel, increasing demand for athletic participation created new opportunities for the development of related running equipment. Consequently, the annual number of patent applications in the field of running protective equipment rose from 11 to 62, representing a 5.63-fold increase. This phase can thus be characterized as a formative stage in the accumulation of technological reserves within the domain of running protective equipment. Since the beginning of the 21st century, the field of running protective equipment has experienced a significant acceleration in technological innovation. This progress has been primarily driven by rapid advancements in foundational applied sciences—such as chemical materials, composite materials, and electronic technologies—alongside an increasing global emphasis on intellectual property protection. Influenced by these technological developments and their interdisciplinary integration, advanced materials including spring-loaded components, polymeric foams, gel inserts, and high-coefficient fiber composites have been extensively incorporated into runnin[illegible]otective gear. These innovations are predominantly reflected in products such as running shoes, leg guards, and knee supports, with the objective of enhancing durability, functional performance, and user safety.

During this period, patent activity within the domain rose substantially, with the annual average reaching approximately 150 applications. Notably, patents related to running protective equipment accounted for 75.40% of all filings during this timeframe. Accordingly, this stage can be characterized as a period of pronounced technological advancement in the development of running protection technologies.

In comparison to the global landscape, China's research and development efforts in the field of running protective equipment commenced at a relatively late stage, with the first patent applications filed in 1987. Since then, the trajectory of Chinese patent filings has remained broadly aligned with international trends. From the perspective of China's share in global patent activity, a clear upward trend has been observed on an annual basis. This growth is closely associated with the effective implementation of national macro-level policy frameworks, including the Outline for Building a Sports Power, Several Opinions on Accelerating the Development of the Sports Industry and Promoting Sports Consumption, and Opinions on Promoting High-Quality Development in the Sports Industry while Encouraging National Fitness and Sports Consumption. Moreover, this pattern reflects the continuous enhancement of innovation capacity and technological competitiveness among Chinese sports equipment manufacturers, particularly within the domain of running protective gear. These advancements are consistent with China's overarching strategy for innovation-driven development.

Regional distribution of patent applications

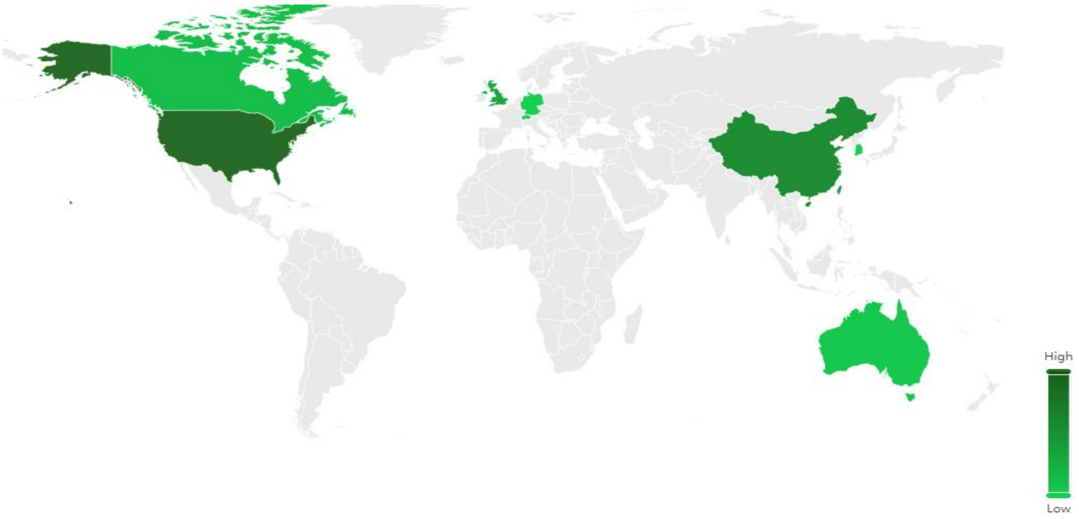

Figure 2 Distributio[illegible]f global patent applications in the field of running protective equipment

The priority country of a patent application refers to the nation in which a specific technology, process, or invention is initially filed for intellectual property protection. In most cases, this country corresponds to the location where the underlying research and development activities are carried out. Accordingly, the designation of a priority country serves as a proxy for evaluating both the intensity of technological activity within a given nation and its contribution to the advancement of related scientific and technological innovation. Figure 2 presents the distribution of priority countries for patent applications in the domain of running protective equipment

[12][13]. The data indicate that the United States—widely recognized as a global leader in both sports and technology—benefits from a vast consumer market and robust research and development capabilities. These advantages have translated into a dominant position in patent activity within this field, with the country holding 1,460 patents, accounting for 33.13% of the global total. China ranks second, with 929 patents, representing 21.08% of worldwide filings. As an emerging economy, China has rapidly developed its intellectual property regime since the establishment of its patent system in 1985. By 2021, it had maintained the highest volume of international patent applications for three consecutive years. In the specific field of running protective equipment, China has achieved significant breakthroughs and technological advancements, contributing to a marked increase in patent output.Other leading countries include Germany (324 patents, 7.35%), Switzerland (243, 5.14%), the United Kingdom (135, 3.06%), Canada (102, 2.31%), Italy (102, 2.31%), Japan (96, 2.18%), the Netherlands (75, 1.70%), and France (62, 1.40%).These figures represent between three and ten entities. From the perspective of national distribution, aside from China, the leading countries prioritizing patent applications in the field of running protective equipment are primarily developed Western nations. This pattern suggests that these countries maintain a technological advantage in this domain, supported by a long-standing accumulation of technical expertise and well-established intellectual property protection systems. Further analysis indicates that patent filings from the top ten priority countries account for approximately 79.66% of the global total. This high level of concentration underscores the dominance of a limited number of technologically advanced nations in driving innovation within the field of running protective equipment.

Global distribution of patent applicants

Table 1 Relevant Information of Global Important Applicants for Running Protective Equipment

sort	Patent right	global Quantity requested	Application share	Applications in the past five years	Last five years Take up a proportion of	nationality
1	Nike	95	2.16%	22	23.16%	USA
2	Cloud company	93	2.11%	17	18.28%	USA
3	Adidas	35	0.79%	2	5.71%	GER
4	Jiangyin Tianguang Technology	22	0.50%	10	45.46%	CHN
5	Anta	17	0.39%	16	94.12%	CHN
6	Fujian Hongxing Erke Sporting Goods	17	0.39%	8	47.06%	CHN
7	Frampton Ellis III	12	0.27%	0	0.00%	USA
8	Puma Europe	12	0.27%	1	8.33%	GER
9	Li Ning (China) Sporting Goods	11	0.25%	7	63.64%	CHN
10	Stange sports protection technology	11	0.25%	6	54.55%	CHN

Table 1 presents statistical data on the top ten high-output patent applicants in the field of running

protective equipment. In terms of application volume, Nike, a cloud-based enterprise, and Adidas AG rank as the top three entities, with 95, 93, and 35 applications, respectively. Notably, the top two positions are occupied by U.S.-based applicants, reflecting a substantial quantitative advantage. This underscores the United States' dominant position in this technological domain, characterized by robust research and development capabilities and considerable technical influence.From a geographical perspective, the distribution of patent applicants comprises three from the United States, two from Germany, and five from China. This distribution suggests that, under national strategies promoting intelligent manufacturing and technological advancement, China's running protective equipment sector has fostered a dynamic innovation ecosystem. Moreover, there is growing momentum among domestic innovators, as evidenced by increased engagement in technological development and heightened awareness of intellectual property rights.However, when assessing the ratio of patent applications to the number of research and development entities, it becomes evident that although China has a larger number of R&D institutions, its patent output remains comparatively low. This discrepancy highlights a gap between innovation potential and the effective transformation of research outcomes into intellectual property assets.The disparity between Chinese entities and leading global corporations in the field of running protective equipment remains significant. This suggests that China currently lacks a dominant enterprise in this domain, thereby highlighting the urgent need to enhance the competitiveness of its innovation-driven actors.A further analysis of patent application shares over the past five years reveals that European and American firms—represented by Nike, a cloud-based company, and Adidas AG—have contributed a relatively small proportion of recent filings. This trend indicates that these well-established corporations have largely completed their foundational technological accumulation in this sector. Consequently, their current investment in research and development related to running protective equipment appears limited, reflecting a reduced level of innovation activity.

In contrast, Chinese companies—such as Tianguang Technology, Anta, and Li Ning—exhibited a relatively high proportion of patent applications during the same period. This indicates that these emerging market enterprises, despite their later entry into the sector, are actively engaged in a phase of technological catch-up. The combined influence of strong policy support and substantial market demand has significantly stimulated both innovation activity and institutional engagement. As a result, these firms have achieved a notable concentration of patent output over the past five years.

IPC technical analysis

Table 2 Top 10 IPC Classification Technologies in the World

Ranking	IPC	Number of patents/pieces	Patents accounted for %	Technical meaning
1	A43B5/06	560	12.71%	Running shoes; Track shoes
2	A43B13/18	537	12.19%	Elastic sole
3	A43B13/14	445	10.10%	Characterized by structural shape
4	A43B13/12	325	7.38%	Multiple layers of soles of different materials

5	A43B5/00	311	7.06%	Sports footwear
6	A63B71/12	170	3.86%	For the body or legs
7	A41D13/05	167	3.79%	It's just a special part of the body
8	A43B13/20	162	3.68%	Inflatable sole
9	A41D13/06	161	3.65%	Knee pads or foot protectors
10	A43B13/04	157	3.56%	Plastic, rubber or vulcanized fiber

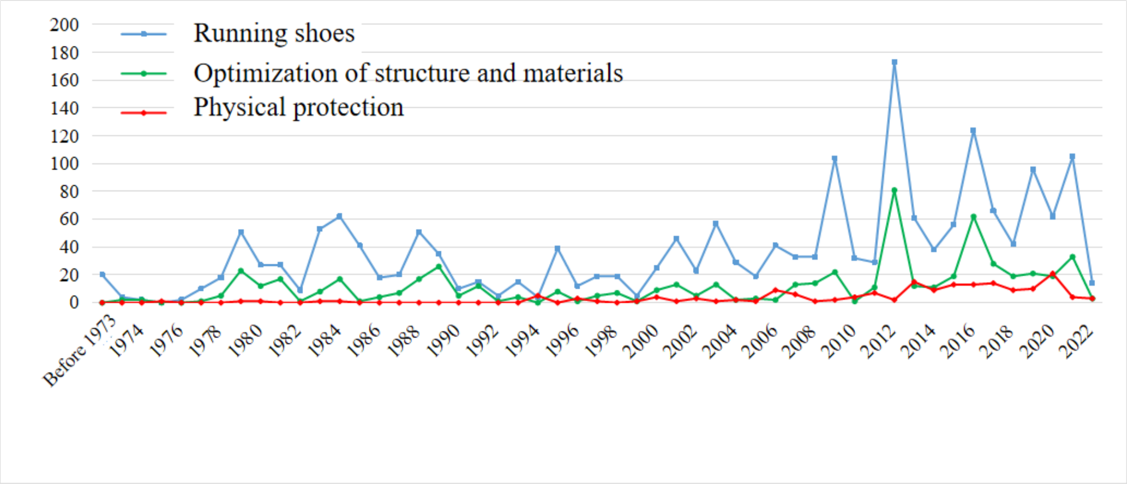

Figure 3 Application trend of key patent technologies in the field of running protection equipment

The International Patent Classification (IPC) system, established by the World Intellectual Property Organization, is based on defined technical criteria and is designed to categorize patents according to their technological content. The structure and distribution of IPC categories provide valuable insights into the technological orientation and developmental trajectory of a given field [14]. In this study, a total of 4,407 patents related to running protective equipment were collected and classified according to IPC codes for statistical analysis. This effort aims to provide a technical reference for the development of patent strategies by enterprises, research institutions, universities, and other innovation-oriented entities operating within this domain. As shown in Table 2, the top ten IPC patent classifications are primarily concentrated in three areas: running shoes, structural and material optimization, and body protection. Among these, patents related to running shoes constitute the largest share, with 1,895 filings, accounting for 42.99% of the overall patent landscape. This dominance is largely attributable to specific technical subclasses, including A43B5/06 (running shoes; track and field shoes), A43B13/18 (elastic soles), A43B13/12 (multi-layer soles composed of different materials), A43B5/00 (sports footwear equipped with non-slip devices, such as cleats), and A43B13/20 (inflatable soles). These findings indicate that, within the domain of running protective equipment, technologies focusing on sole-based shock absorption and anti-slip functionality represent a major area of technological concentration.The category pertaining to structural and material optimization encompasses 602 patents, representing 13.66% of total filings. This group is primarily characterized by technologies classified under A43B13/14 (defined by structural shape) and A43B13/04 (comprising plastic, rubber, or

vulcanized fiber), along with other related subclasses.Additionally, the body protection category includes 498 patents, accounting for 11.3% of the total. This segment mainly comprises technologies classified under A63B71/12 (protective devices for the body or legs), A41D13/05 (garments designed for the protection of specific body parts), and A41D13/06 (knee pads and foot protection), along with other associated technical classifications.

From the perspective of IPC technology development trends, the three principal technical domains—running shoes, structural and material optimization, and body protection—have demonstrated a relatively consistent trajectory in patent application growth. Prior to 2000, these areas were in a phase of technological latency, characterized by sporadic filings and the absence of a discernible development trend. However, after 2000, they transitioned into a stage of technological maturity, marked by a significant acceleration in patent output. During this period, patent applications followed a generally upward yet fluctuating trajectory. Notably, a total of 1,878 applications were filed across the three domains, accounting for 42.61% of all patents submitted. This shift signifies the onset of a "golden age" for technological advancement in the field of running protective equipment.From a comparative perspective, both the cumulative volume and growth rate of patents related to running shoes significantly exceed those in structural and material optimization, as well as body protection. This indicates that running shoes constitute a primary research focus within the broader technological landscape of running protective equipment.Furthermore, this trend indicates that technological competition among innovative actors is primarily concentrated in the domain of running shoes. The underlying rationale is that modern running shoes have become essential athletic equipment, embodying a sophisticated integration of engineering and technology. Their structural features—such as support, cushioning, and elasticity—play a critical role in mitigating ankle injuries among runners. Concurrently, the growing popularity of running, coupled with continuous advancements in shoe functionality, has significantly intensified consumer demand for high-performance footwear. In response to these market dynamics, numerous sports equipment manufacturers are actively investing in research and development to enhance running shoe technologies, increase market share, and maximize economic returns.

Analysis of patent application types and validity

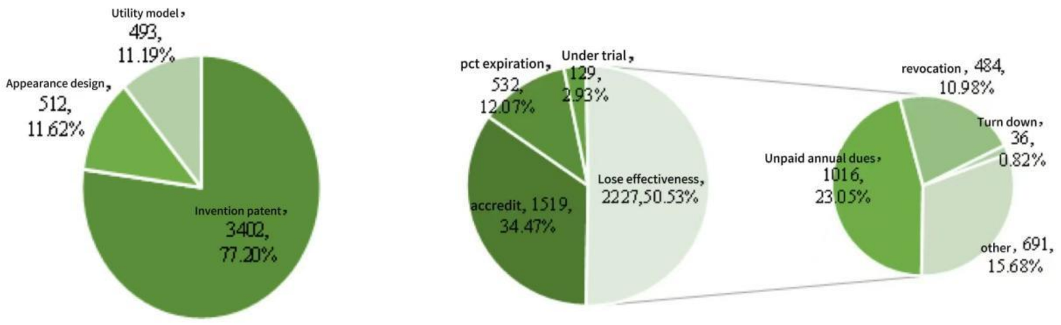

Figure 4 Patent types and legal documents in the field of global running protective equipment

In view of the strong research and development momentum demonstrated by numerous enterprises in the field of running protective equipment—coupled with significant technological advancements—it is essential to examine both the types and legal statuses of existing patents. Such analysis provides critical reference value for the formulation of effective patent strategies within the industry [15].Figure 4 presents a comprehensive overview of patent classifications and their legal statuses related to running protective equipment. Among the various categories, invention patents dominate the landscape, with 3,402 applications accounting for 77.20% of the total. This is followed by design patents and utility models, with 512 and 493 applications respectively, representing 11.62% and 11.19%. These figures indicate that invention patents serve as the primary form of intellectual property protection in this field, highlighting the significance of running protective equipment in safeguarding athletes' health and preventing running-related injuries.Moreover, the manufacturing of such equipment is subject to rigorous quality standards and technical specifications, thereby necessitating a high degree of innovation in R&D activities. Simultaneously, due to the geographic concentration of core technologies and the proprietary nature of enterprise-level expertise, it is imperative for R&D personnel to overcome patent barriers through sustained technological innovation. As a result, invention patents continue to represent the predominant form of protection within the patent landscape of this sector.

From the perspective of legal status, a total of 1,519 patents in the field of running protective equipment are currently authorized and valid, accounting for 34.47% of all filings. In contrast, 2,227 patents are classified as invalid, representing 50.53% of the total. These figures suggest that the overall quality of patents in this domain remains relatively low, indicating limited market applicability and weak technological durability.Further analysis reveals that unpaid annual fees account for 23.05% of the invalid patents, while patent withdrawals constitute 10.98%. This highlights a significant imbalance between the intellectual property revenues generated by many patents in the sector and the costs required for their maintenance. Challenges related to commercialization and insufficient economic returns have contributed to the invalidation of a substantial number of patents due to non-payment of renewal fees or voluntary withdrawal.

Analysis of patent technology evolution

Core patent screening

Core patent screening indicators

As a fundamental aspect of patent analysis, the identification and extraction of core patents are essential for uncovering key technologies within the target domain. This process plays a pivotal role in elucidating the technological evolution of the field [16]. Drawing upon the methodological frameworks established by Li Meining [17], Xu Xin [18], and Wang Zhixing [19], this study develops a core patent evaluation index system encompassing the dimensions of technological advancement, application potential, and operational stability, as illustrated in Table 3.To ensure a scientifically rigorous assessment and accurately identify high-quality patent samples, the evaluation framework incorporates both subjective and objective factors. A hybrid-weighted

TOPSIS model is employed to assign index weights and calculate composite scores for patents within the domain of running protective equipment.

Table 3 Core patent screening index system

Primary index	Secondary index	weight
The advanced nature of patented technology	Number of patent citations	0.11
	Cited frequency	0.09
	Number of patentees	0.18
Patent application prospect	Number of IPC class numbers	0.17
	Quantity of claim	0.09
	Number of patents in the same family	0.14
Patent operation stability	Number of pledges, transfers and licenses	0.07
	Survival time	0.08
	Manual pages	0.07

Analysis of core patent technology

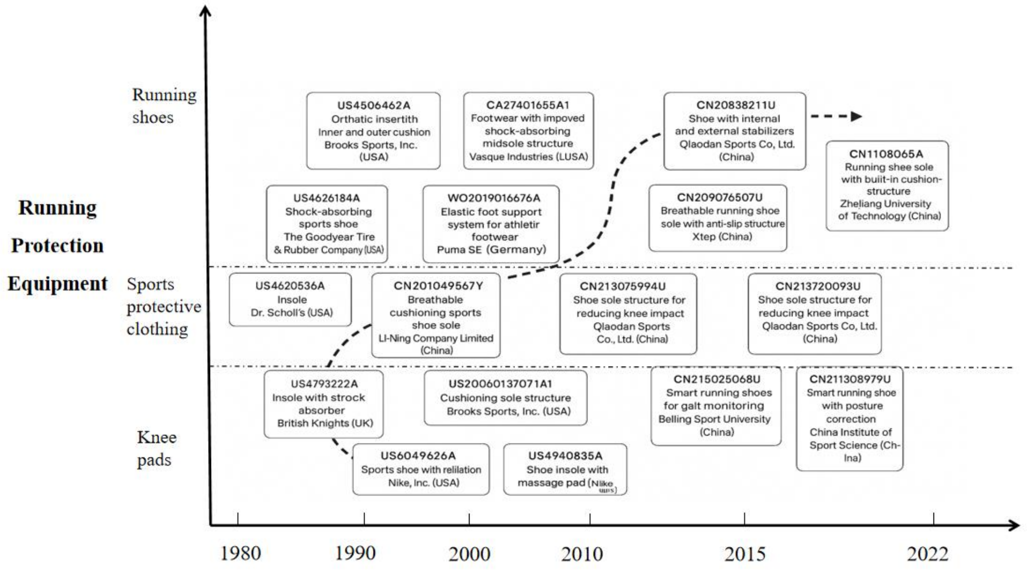

Figure 5 Technical Evolution of Running Protection Equipment

As illustrated in Figure 5, the technological evolution within the domain of running protective equipment primarily centers on three key components: running shoes, sports protective apparel, and knee pads. This evolution encompasses a range of technological innovations, including sole stabilization plates, compressible midsole materials, shoe body support systems, garment material optimization techniques, advancements in stitching processes, the integration of protective elements into garments, innovative structural designs for knee pads, joint fabrication methods, fitting and fastening mechanisms, and optimizations of shock-absorbing devices.

Technical evolution analysis of running shoes

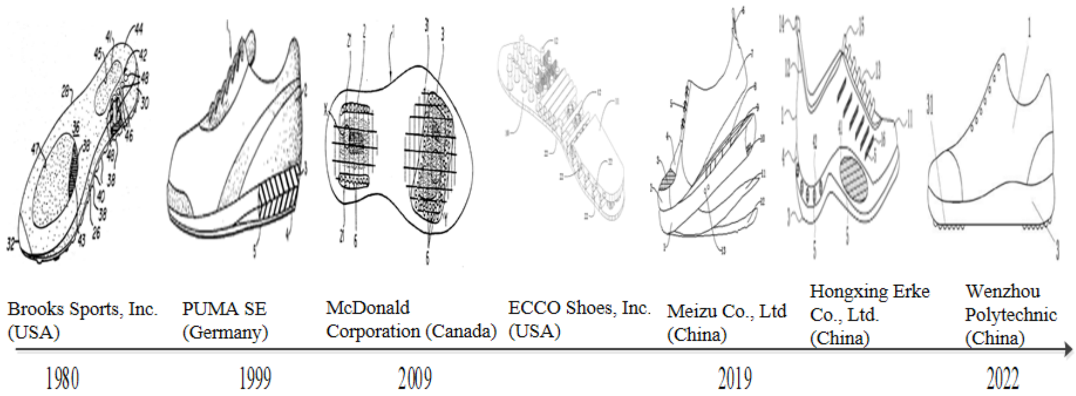

Figure 6 Technical Evolution of Running Shoes

As illustrated in Figure 6, the evolution of patented technologies for running shoes primarily centers on three key areas: stability mechanisms, sole support and protection structures, and the diversification of safety functionalities. Specifically, these advancements encompass a range of technical domains, including sole stabilization plates, compressible midsole materials, structural support systems for the shoe body, elastic components, internal shock absorption systems, dehumidification mechanisms, enhancements in breathability, optimization of antibacterial properties, and integrated upgrades to safety devices within the overall shoe design.

Due to the forward-leaning posture of runners and the inherent instability of the sole during motion, excessive pronation frequently occurs in the knees and feet. If left uncorrected, this condition may lead to foot bone deformities, knee varus, osteoarthritis, and other related musculoskeletal disorders. In response, early innovations in running shoe design primarily focused on enhancing sole stability mechanisms to regulate the rotational movement of the foot and knee.

Brooks Sports Co., Ltd. (United States) developed a sole stabilization device featuring a dual-layer cushioning structure. This design incorporates a primary buffer layer composed of an elastic material with a Shore hardness rating of 35, strategically positioned in the forefoot, arch, and heel regions to provide targeted shock absorption and biomechanical support.Additionally, an opening is incorporated at the junction between the heel and the sole, within which a secondary buffer layer—composed of an elastic material with a Shore hardness of 40—is embedded. The dual-layer cushioning design of this stability device effectively absorbs the mechanical stresses generated during running. This innovation mitigates impact forces between the foot and the shoe body, enabling the footwear to better withstand lateral forces experienced by the lower limbs. Moreover, it plays a critical role in limiting excessive rotational motion of both the foot and knee.Building upon this concept, Puma (Germany) has integrated an elastic cushioning material with a Shore hardness of 60–65, along with non-porous rubber, into the midsole structure. This enhancement is intended to further improve shock attenuation and increase sole stability. To optimize the regulation of foot and knee rotation, the width of this sandwich-structured insert exceeds two-thirds of the total sole width. The insert is carefully engineered to extend across both the heel and the medial side of the upper. This configuration significantly reduces both the

amplitude and frequency of lateral heel displacement, thereby preventing excessive pronation and promoting proper alignment of the foot and knee.

With improvements in the fundamental performance characteristics of running shoes, efforts to enhance durability, comfort, and protective functionality have increasingly focused on optimizing sole support and protection systems. During running, the impact forces and pressure generated by lower limb movement are primary factors contributing to both footwear degradation and injury risk.In response to these challenges, McDonald Company (Canada) has developed a multi-structure support system for footwear, based on the principle of fluid transfer. This system incorporates multiple elastic structural components, micro air cushions, and compression elements embedded with buoyancy magnets. As a result, the shoe exhibits excellent compression resistance and shock absorption performance. Under this system, the impact forces and kinetic energy generated during running can be effectively absorbed and dissipated, thereby significantly reducing injury risk while enhancing the structural durability of the shoe.However, as advancements in basic performance characteristics continue, the integration of numerous functional elements has led to a substantial increase in shoe weight, along with considerable spatial compression, thereby limiting foot mobility.As a result, this not only reduces flexibility and comfort but also increases the risk of injuries to various parts of the foot, including the toes, arches, and heels. To address this issue, Aibu Shoes Company (United States) conducted a comprehensive study on the physiological structure of the human foot. Guided by the principles of product simplification and functional optimization, the company adopted an integrated and lightweight shoe desi[illegible]n. By incorporating a combination of specialized low-density PU variants (with a density of 0.35 g/cm^3), EVA polymers, and NBR rubber materials, the design effectively balances the essential structural performance of the shoe body with enhanced comfort and flexibility.

Driven by rapid advancements in materials science, intelligent equipment, and manufacturing technologies, the safety performance of running shoes has undergone comprehensive enhancement and expansion. Influenced by factors such as extended working hours, irregular rest patterns, and the pursuit of exercise efficiency, there is a growing trend among runners favoring evening workouts. In response to this development, Zhangzhou Meizu Industrial Design Co., Ltd. (China) has introduced a fiber-optic illuminated running shoe that leverages existing technologies to enhance user safety during nighttime activities. The design incorporates a light-emitting element and a reflective structure positioned at the heel of the sole to improve the shoe’s luminous performance. The implementation of fiber-optic lighting effectively addresses common issues found in conventional products, such as unstable illumination, excessive heat generation, and limited durability.Beyond enhancing the functional utility of running shoes, this innovation significantly reduces the risk of impact-related injuries during nighttime exercise. Furthermore, to tackle persistent challenges related to poor breathability and inadequate moisture management in existing running shoes, Hongxing Erke Co., Ltd. (China) has achieved notable progress by advancing upper material technologies and overcoming the limitations of traditional manufacturing processes.This innovation has enabled the company to enhance both air permeability and dehumidification performance by incorporating an active ventilation system along with a dual-layer breathable structure within the shoe upper. The active ventilation system primarily consists of a temperature and humidity sensor, micro air pump, airbag, inlet and outlet tubes, ventilation apertures, and one-way intake and exhaust valves. It is designed to automatically monitor the internal temperature and humidity of the shoe. When these parameters exceed

predefined thresholds, the system actively expels heat and moisture, thereby maintaining a consistently dry and comfortable internal environment.To address the risk of bacterial infections resulting from prolonged physical activity and to safeguard foot health, Wenzhou Vocational and Technical College (China) has implemented a targeted strategy to regulate internal humidity and suppress microbial growth within footwear. This approach involves integrating antibacterial and dehumidifying interlayers into the sole. The composite structure primarily comprises bactericidal agents and desiccants, housed within an internal mounting slot that allows for convenient replacement. This design ensures sustained effectiveness in both antibacterial protection and moisture control within running shoes.

Evolution analysis of sports protective clothing technology

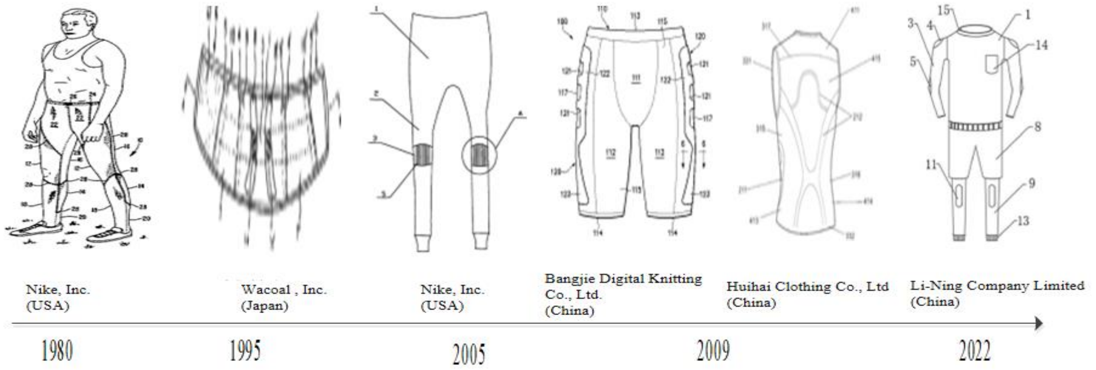

Figure 7 Technical Evolution of Sports Protective Clothing

Figure 7 illustrates the evolution of patented technologies in the domain of sportswear. Within the field of sports clothing technology, the development of key patents has primarily focused on several critical areas, including thermal regulation and insulation, abrasion resistance, knee and waist protection, muscle strain mitigation, and shock absorption. These advancements encompass the refinement of fabric materials, improvements in stitching techniques, the integration of protective components into garments, and the implementation of innovative structural designs in apparel construction.

The initial advancements in sportswear technology primarily focused on enhancing the thermal regulation and abrasion resistance properties of garments. It is well established that runners generate substantial heat during exercise. Given the temperature sensitivity of muscles in an active state and the variability of environmental conditions, maintaining a stable body temperature is essential. This thermal stability helps prevent muscle spasms, stiffness, and other symptoms affecting muscles, tendons, and surrounding soft tissues that may result from exposure to excessively cold or hot environments.To address this issue, Nike (United States) has developed a line of sports protective clothing. The outer layer of this apparel is made from a specialized mesh material designed to facilitate efficient heat dissipation. Simultaneously, a thermostatic component is integrated into muscle-sensitive areas using four-needle flat-seam technology. This component comprises lightweight composite materials—such as wool and knitted spandex—that offer effective ventilation and thermal insulation.To reduce friction-related injuries that runners may

sustain during falls, WACO Company (Japan) has developed sportswear incorporating protective coatings and elastic components. These garments integrate polymer materials including polyamide, polyester, polyurethane film, and polytetrafluoroethylene (PTFE) on their outer surfaces. This design significantly enhances both abrasion resistance and impact-buffering performance in sports apparel.

With the continuous advancement of sports protective clothing, addressing issues such as knee injuries and lumbar strain—common occurrences during running—has become a primary focus. Consequently, research and development in this field increasingly emphasize enhancing protective functionality for both the knees and waist. While maintaining comfort and a lightweight design as fundamental principles, Nike (United States) has developed a type of waistband-integrated sportswear made from stretchable woven fabric. This garment features a raised, contoured fit that conforms to the abdomen and waist. In addition, it incorporates elastic resin-based bone braces positioned along both sides of the waist and spine. These braces are specifically engineered to expand in response to changes in abdominal pressure during physical activity, thereby effectively stabilizing the lumbar spine and alleviating fatigue or pain in the lower back.To simultaneously protect the knees while maintaining the comfort typically associated with traditional sweatpants, Zhejiang Jiejie Digital Knitwear Co., Ltd. (China) has developed a pair of sweatpants that integrates both comfort and knee-support functionality. The pants incorporate a knee brace component featuring a groove anatomically tailored to fit the outer contour of the human knee. This design minimizes friction between the knee brace and the lower limb, reducing discomfort caused by close contact between the fabric and the runner's skin. As a result, it effectively prevents potential knee injuries and improves overall wearing comfort.

Amid growing concerns over sports-related injuries and heightened public awareness of health, consumer expectations for sportswear have evolved beyond the localized protection of areas such as the knees and lumbar spine. These expectations now encompass comprehensive protection that targets multiple regions of the human body. In response, the Chinese sportswear company Li Ning has introduced an innovative reinforced bodysuit. This form-fitting garment employs a four-stitch, six-thread sewing technique to integrate hollow, breathable mesh fabric—composed of spandex and nylon—into the lower back and outer thigh areas. By applying compression principles, the design incorporates laser-cut laminated fabrics and thermoplastic (TP) adhesive cloth, which are strategically positioned over key muscle regions, including the gluteal area, lateral thigh muscles, abdominal muscles, and back muscles.This design significantly reduces the risk of strain in critical muscle groups commonly engaged during running, such as the rectus abdominis, trapezius, gluteus maximus, semimembranosus, and semitendinosus. To minimize the costs associated with acquiring multiple pieces of protective gear and to diversify functional coverage, Yangzhou Huihai Clothing Co., Ltd. (China) has developed an integrated solution based on modular connection principles. This approach employs mechanical, detachable, and electrical connection methods to embed shock-absorbing silicone pads into garments. These pads are strategically positioned in high-impact areas, including the shoulders, elbows, back, and knees, providing simultaneous protection to multiple vulnerable body parts. This innovation effectively addresses the challenge faced by runners who would otherwise need to purchase separate protective equipment for each individual region.

Evolution analysis of knee brace technology

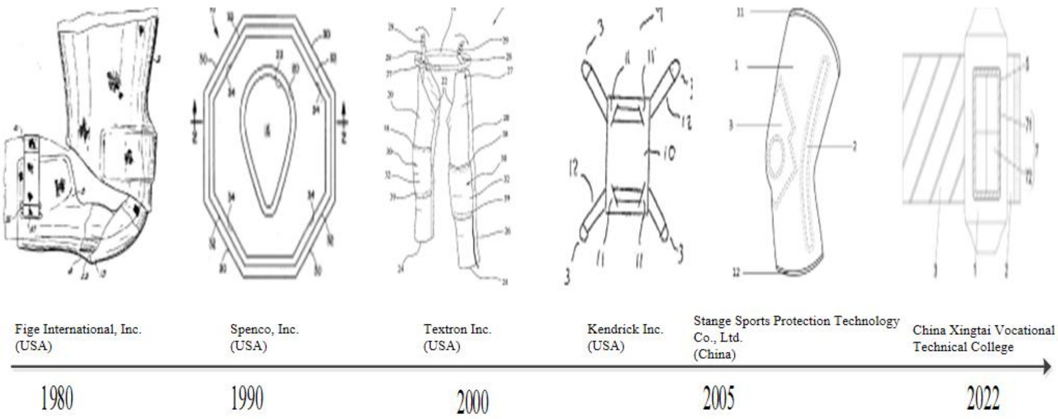

Figure 8 Evolution Diagram of Knee Support Technology

Figure 8 illustrates the evolution of patented technology in the domain of knee pads. The progression of key patents within this field primarily focuses on three core aspects: abrasion and slip resistance, ergonomic comfort, and the enhancement of protective performance. These advancements encompass a variety of technologies, including structural design, joint articulation techniques, fastening and stabilization mechanisms, optimization of cushioning systems, and the development of impact-resistant materials.

In the initial applications of knee pads, functional limitations were evident due to coarse surface friction treatments and inadequate fixation mechanisms, resulting in frequent slippage and displacement among runners. To address these issues, Fige International Company (United States) developed an innovative knee brace featuring a double-layer structure with a specialized pad arranged in an overlapping composite configuration. This pad is composed of a soft, closed-cell rubber foam known for its excellent frictional properties and flexibility. During repeated knee flexion, the pad maintains stable contact between the knee and the brace, effectively preventing slippage or detachment.Subsequently, Spenco Company (United States) introduced an advanced knee pad aimed at further improving stability through refinement of the existing design. Retaining the double-layer structure, the new knee pad was optimized based on the physiological characteristics of the human knee. In particular, modifications to the external contour significantly enhance anatomical conformity, resulting in a more precise fit and increased stability during use.

With the enhancement of the fundamental functions of knee pads, there is increasing demand for improved wearing comfort and the mitigation of issues such as poor blood circulation and skin abrasion caused by excessive compression. In response to this need, Textron has introduced an innovative type of knee pad. The primary structure consists of flexible materials that conform to human leg anatomy, including nylon mesh, polyester film plastic, and leather. Departing from traditional fixation methods that rely on cloth straps wrapped around the legs, the new design incorporates openings at both the upper and lower sections of the knee pad. Flexible carabiner rings are integrated into these openings, enabling a secure fit without the need for tightly fastened

straps. This approach effectively alleviates the discomfort experienced by runners due to excessive pressure on the knees and legs caused by conventional strapping techniques.Furthermore, to enhance wearing comfort even further, Kendrick Company (United States) has developed a flexible bow pad integrated into the knee pad. This component is constructed from neoprene and plastic foam fabric, offering improved tactile performance and anatomical conformity. Additionally, its distinctive attachment method—using a specially designed elastic woven belt—ensures that the pad maintains alignment with the runner's movements. As a result, this design not only enhances comfort but also effectively addresses displacement and slippage, which are common problems associated with traditional knee braces.

With the rapid increase in the number of runners and the diversity in their physical fitness, running form, and training volume, knee injuries of varying severity have become increasingly common. This trend has prompted a heightened focus on improving the protective functions of knee pads in technical development. In response to this demand, China Stange Sports Protection Technology Company has designed an innovative sports knee guard. This product primarily consists of three components: a cylindrical sleeve woven from elastic fibers, a support strip constructed from flat spring material, and a massage pad made of soft silicone. These elements are integrated using advanced hot-press cloth bonding technology.During use, the double-fiber cylindrical sleeve provides enhanced compressive support, improving adherence between the knee brace and the knee joint. The support strip, composed of flat spring material, generates elastic resistance during deep knee flexion, offering superior cushioning performance and reducing the risk of strain. Furthermore, the triangular soft silicone massage pad delivers therapeutic stimulation to support quadriceps muscle health and acts as a buffer against collisions and impacts at the knee joint, thereby protecting critical anatomical structures such as the patella.Through the synergistic integration of these three elements, the protective efficacy of the knee brace has been significantly enhanced, effectively combining both preventive and rehabilitative functions.

Conclusions and countermeasures

Conclusion

Based on data from the Patsnap global patent database, this study analyzes patents related to running protective equipment as the primary sample. Employing a patent bibliometric methodology, it examines the technological development trends in this domain from multiple perspectives, including application trends, geographical distribution, applicant profiles, IPC technology classifications, patent types, legal status, and pathways of technological evolution. The key research findings are summarized as follows:

(1) The patent applications for global running protective equipment technology exhibit distinct stages of technological development. The period from 1898 to 1970 represents the embryonic stage, characterized by sporadic and infrequent patent activity, reflecting the initial phase of innovation in this field. From 1971 to 1999, a developmental phase emerged, driven by the growing popularity of running events and increased public participation in sports, which created substantial demand for related protective equipment and stimulated technological advancement. After 2000, the field entered a stage of rapid innovation, supported by significant progress in

foundational applied sciences and heightened global awareness of intellectual property rights. This progression has elevated the domain of running protective equipment to a new level of technological sophistication and innovation capacity.

(2) Global patent applicants for protective equipment technology are predominantly concentrated in a limited number of countries, primarily the United States, China, and Germany. Western developed nations maintain a leading position in this technological domain. A significant disparity exists in both the volume and impact of patent applicants, with the United States holding a clear and sustained technological advantage. Although China entered the field at a relatively late stage, its innovation capacity and technological influence have shown steady growth, indicating considerable potential for future advancement.

(3) The global patent landscape for running protective equipment is predominantly composed of invention patents. However, a clear imbalance exists between the revenue generated from intellectual property and the corresponding maintenance costs. This disparity poses significant challenges to the commercialization of such patents and undermines their economic viability. As a result, many patents are ultimately abandoned due to non-payment of annual maintenance fees.

(4) Technical research and development in the field of running protective equipment primarily focuses on three key areas: running shoes, sports protective clothing, and knee pads. R&D efforts related to running shoes emphasize stability mechanisms within the footwear, protective structures supporting the sole, and the integration of diverse safety features. Notably, current technical hotspots include sole stability plates, compressible sandwich materials for soles, and support systems within the shoe body.In the domain of sports apparel, major technological focuses include temperature regulation and insulation, anti-friction properties, knee and waist support, prevention of muscle strains, and shock absorption. Key development directions involve fabric material optimization, advancements in stitching techniques, and the integration of protective elements.Knee pad technology primarily aims to improve wear resistance and slip prevention while ensuring user comfort. Research in this area concentrates on structural design innovations, joint articulation methods, fastening and securing mechanisms, optimization of cushioning systems, and the enhancement of impact-resistant materials. These aspects represent major focal points for ongoing technological development in this domain.

Suggestions

Based on the aforementioned research findings, this paper aims to promote the development of China's running protective equipment industry by fostering technological innovation and enhancing strategic patent deployment among enterprises in the sector. Accordingly, the following countermeasures and policy recommendations are proposed:

(1) The delayed establishment of an effective intellectual property (IP) protection system in China, coupled with limited administrative experience, has resulted in several long-standing issues. These include an underdeveloped legal framework, inconsistent enforcement mechanisms, inadequate enterprise capacity to resolve IP disputes, and the generally low quality of intellectual property assets. Such challenges hinder not only the high-quality development of China's protective equipment industry but also significantly restrict enterprise-led research, development, and innovation.To address these issues, it is necessary for the government to first reinforce the legal foundation of IP protection by promoting coordinated amendments to the Patent Law

Trademark Law, and Copyright Law. This effort should also involve standardizing procedures for IP litigation and increasing penalties for infringement and non-compliance. Second, the patent examination process and the broader intellectual property evaluation system must be further improved. While it is important to protect low-value patents and raise the overall quality of patent portfolios, it is equally essential to prevent the misuse or abuse of IP rights by enterprises seeking to exploit regulatory loopholes [20].Finally, a national early warning and emergency response mechanism for managing IP risks should be established. This includes enhancing foreign-related IP risk prevention and control systems, encouraging enterprises to actively participate in international IP competition, contribute to the formulation of global technical standards, and expand transnational patent applications. Collectively, these measures will help safeguard the legitimate rights and interests of Chinese enterprises in overseas intellectual property matters.

(2) Fostering innovative leading enterprises is essential for enhancing the competitiveness and technological influence of China's protective equipment industry. In comparison to developed economies, the lack of such enterprises in China significantly undermines its international standing in this sector. Enterprises serve as a dynamic force in driving innovation, with leading firms functioning as the cornerstone of scientific and technological advancement. These entities have the capacity to aggregate and integrate critical innovation resources—including talent, capital, and technology—while serving as service platforms, incubators, and accelerators through their demonstration and multiplier effects.To address this gap, the government should intensify policy support and increase financial investment. On the one hand, relevant administrative departments must implement preferential policies such as tax deductions for R&D expenditures and accelerated depreciation of research equipment. These measures aim to unlock the innovation potential of enterprises and promote sustainable development by alleviating financial burdens. On the other hand, government procurement mechanisms should be leveraged to create demand-driven markets. For example, direct procurement of protective equipment and support for consortia composed of large enterprises and small or micro-sized firms can stimulate industry growth and participation in public procurement projects [21].Secondly, it is crucial to optimize financing channels to reduce operating costs and enhance innovation efficiency. This includes encouraging economies of scale, attracting angel investment, and advancing the "venture capital + incubation" model. Moreover, it is necessary to provide professional and efficient financial services, technological platforms, and business support for both scientific R&D and the commercialization of research outcomes.Finally, the government should establish a streamlined mechanism for attracting high-level talent to the sector. This includes improving welfare and security systems, promoting university–industry collaboration in talent development, and supporting flexible employment models, such as part-time work or entrepreneurial activities among researchers affiliated with academic and research institutions.

(3) Given current trends in the evolution of patents related to running protective equipment, it is imperative for Chinese enterprises to strengthen their awareness of intellectual property (IP) protection and adopt strategically aligned patent management practices. The formulation of effective patent strategies must integrate internal innovation capacity with external environmental dynamics to support sustainable industrial growth [22].In the early stages of technological development, enterprises should prioritize the accumulation of foundational technologies through a combination of approaches, including technology licensing, acquisition of high-value patents, and targeted talent recruitment. Concurrently, R&D investments should be significantly increased

in critical segments such as running shoes, sports apparel, knee pads, and adjacent technologies to ensure precise alignment with emerging technological frontiers.Moreover, it is essential to proactively address technical barriers and mitigate the risk of patent infringement, particularly in response to challenges posed by foreign competitors. This requires ongoing exploration of alternative technological trajectories and novel research directions within the domain of running protective equipment.Ultimately, the establishment of a robust core patent protection network will enable enterprises to secure sustainable competitive advantages and advance toward strategic leadership within the global marketplace.

(4) Enhancing technological innovation within enterprises and increasing the market value of patent outputs are critical objectives for improving the overall quality and effectiveness of intellectual property in the protective equipment sector. The widespread prevalence of low-quality patents and the resulting lack of economic returns presents substantial challenges. The emergence of "junk patents" not only wastes essential innovation resources—such as talent, time, and capital—but also distorts the concept of innovation by promoting utilitarian approaches that lack true technological value [23].To address these challenges, enterprises in China's running protective equipment industry should adopt innovation strategies that emphasize the role of technology in driving industrial development. It is essential to integrate scientific research with patent-oriented R&D, guided by market demand. Efforts should focus on identifying technological hotspots and value-driven research directions, while reasonably increasing investment in targeted research and development.Furthermore, adopting a collaborative model that links industry, academia, and research institutions is a vital mechanism for progress. By capitalizing on the advantages of team-based cooperation, enterprises can actively pursue breakthroughs in core technologies and accelerate the commercialization of research achievements in the field. The ultimate goal is to develop high-quality running protective equipment that meets both market demand and intellectual property standards.In conclusion, this approach aims to transform and upgrade innovation dynamics across Chinese enterprises in the sector, fostering sustained and high-quality development.

Acknowledgments

The authors are grateful to the Staff members of Physical Education Department of Wuxi Taihu University

Reference

1. Yin Ding. Analysis of patent information of Chinese sports brand enterprises [J]. Sports Culture Guide,2014(10):118-121+172.
2. Li Ming. The causes of "low-end lock-in" of China's manufacturing global value chain and its breakthrough strategy [J]. Quotient,2015(38):101.
3. Wang Chenxi, Man Jianghong. Connotation, logic and realization path of high-quality development of sports industry under the "double cycle" pattern [J/OL]. Journal of Shenyang Institute of Physical Education :1-8[2022-11-29].
4. Wang Junhan, Li Rongri. The development concept of sports industry under the new development Pattern: Value appeal, realistic review and promotion strategy [J]. Sports Culture Guide,2022(07):58-64.
5. Mother Yingxiu. Construction of physical motor Function evaluation index System of young middle and long distance Runners and its empirical study on lower extremity sports injury [D]. Capital Institute of Physical Education, 2022.
6. WANG Shikui. Research on running protective sports pants based on biomechanics [D]. Shanghai University of Engineering Science and Technology, 2015.
7. Cavalheiro Mariana Brandao,Cavalheiro Gabriel Marcuzzo do Canto,Mayer Verônica Feder,Marques Osiris Ricardo Bezerra. Applying patent analytics to understand technological trends of smart tourism destinations[J]. Technology Analysis & Strategic Management,2021,33(9):1101-1117.
8. WANG Jun.Dynamic Research on the evolution and distribution pattern of international sporting goods technology -- Taking football and its related patented technology as an example [J]. Journal of Chengdu University of Physical Education, 2013, 39(8): 11-16.
9. Ming Yu, Si Huke. A Comparative study on patent R&D of sports shoes between Chinese sports brand manufacturers and Nike [J]. Journal of Wuhan University of Physical Education, 2013, 47(9): 35-40.
10. Yin Long, Li Fang, Wang Lei, Si Huke. Competitive Intelligence Analysis of global Sports equipment Technology patents: A case study of Golf club technology patents [J]. Journal of Capital Institute of Physical Education, 2016, 28(3): 258-264.
11. Wan Yu, Zhang Yuanliang. Research on Competitive intelligence of international sports equipment patent Technology [J]. Journal of Xi 'an University of Physical Education, 2018, 35(5): 521-531.
12. Yuan Xinyue. Research on Technological Innovation Strategy of LNG ship Enterprises based on Patent data mining [D]. Jiangsu University of Science and Technology, 2021.
13. FU Honghao, Guo Zhen, Liu Bo, ZHANG Tingan, Gong Bo. Research on International Competition Situation and Core Technology Application of patents in football related Fields: Based on Innography data analysis platform and national perspective [J]. China Sports Science and Technology, 2022, 58(4): 40-51.
14. Alok Khode, Sagar Jambhorkar. Augmenting keyword-based patent prior art search using

weighted classification code hierarchies[J]. International Journal of Business Intelligence and Data Mining,2022,21(4):397-418.

15. Yin Zhang,Yongzhong Qiao. Analysis of the Patent Information of SiC Ceramics Granted by China[J]. IOP Conference Series: Materials Science and Engineering,2018,381(1).
16. Wu Jie, Yuan Xinyue. Development of BOG treatment technology for LNG carriers [J]. Ship Engineering, 2020, 42(4): 21-25.
17. Li Mei-ning. Research on Comprehensive Evaluation of Technology prediction based on TOPSIS Patent Evaluation Model under Market environment [J]. Information Engineering, 2020, 6(5): 53-65.
18. Xu Xin, ZHAO Wenhua, YAO Zhanlei. Research on high-quality patent recognition and its application from multi-dimensional perspective [J]. Modern Information, 2019, 39(11): 13-22.
19. Wang Zhixing, Fu Qinwei, Du Qijie. Construction of patent strength evaluation model based on Delphi Method [J]. Enterprise Science & Technology Development, 2019(6): 132-135.
20. Song Yagang. Research on Evaluation of technological innovation ability of Chinese sporting goods Enterprises based on Patent perspective [D]. Shanghai University of Sport, 2017.
21. He Bing, Zhou Liangjun, Chen Xiaoying, Xin Li. Theoretical and empirical research on the international competitiveness of China's sporting goods industry [J]. Sports Science, 2007, (7): 14-22.
22. Cao Yong, Zhao Li. Research on the Mechanism of patent acquisition, patent protection, patent commercialization and technological innovation performance [J]. Science Research Management, 2013, 34(8): 42-52.
23. Bao Fangfang, Qiao Fengjie. Research on patents of foreign sporting goods enterprises in China [J]. Sports Culture Guide, 2015, (1): 101-103.

数字 Click here to access/download;Figure;figures.docx

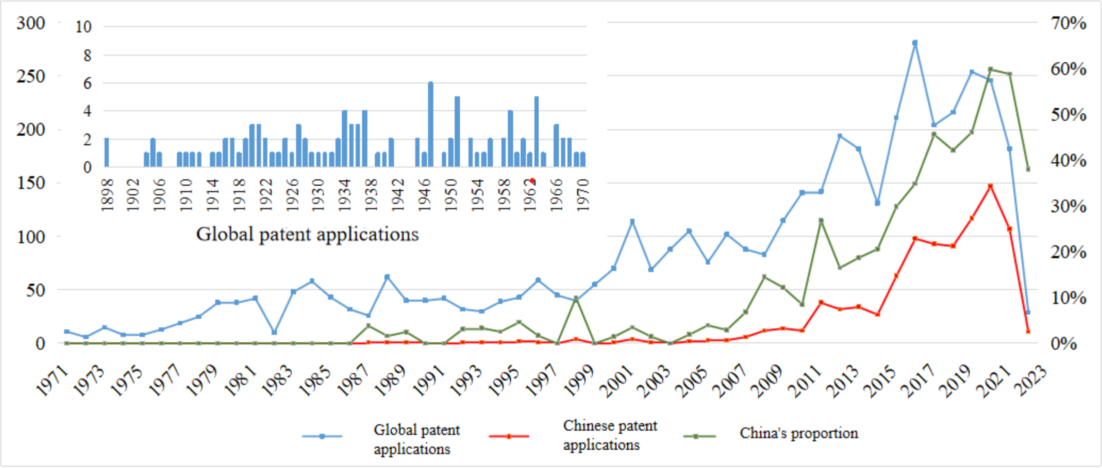

Figure 1 Trend of global patent application in the field of running protective equipment

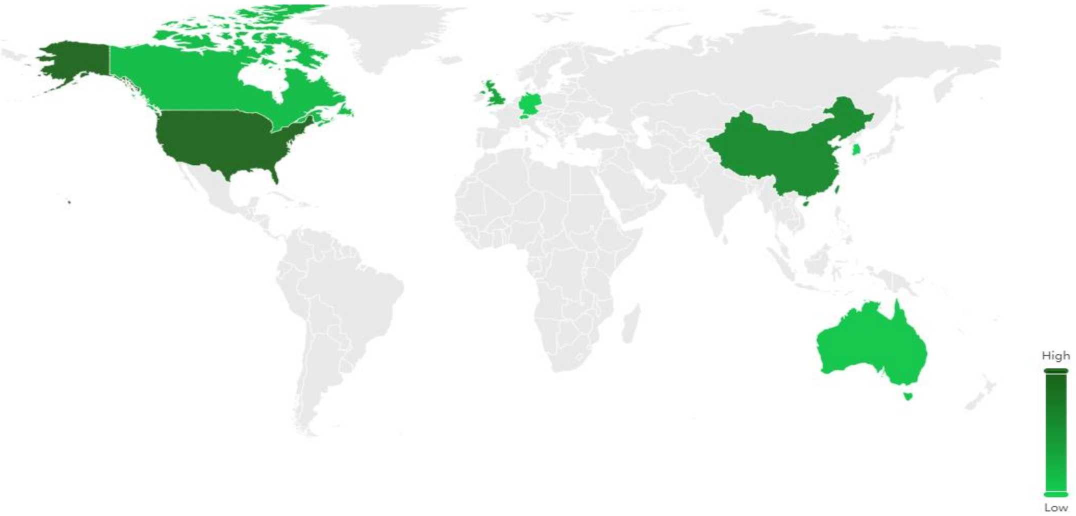

Figure 2 Distribution of global patent applications in the field of running protective equipment

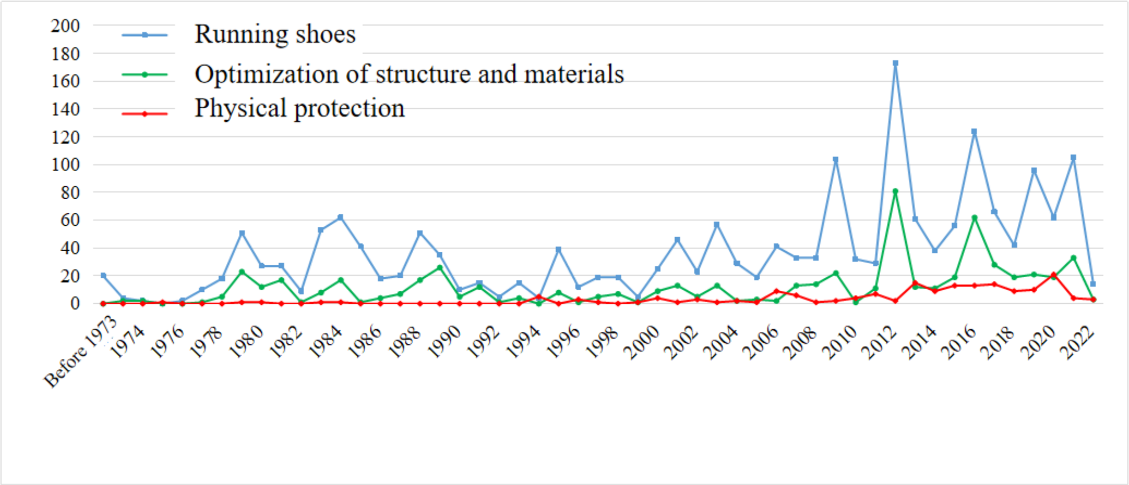

Figure 3 Application trend of key patent technologies in the field of running protection equipment

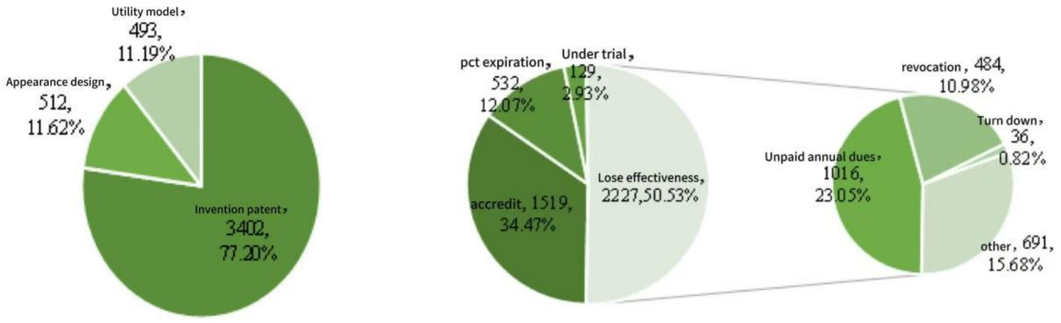

Figure 4 Patent types and legal documents in the field of global running protective equipment

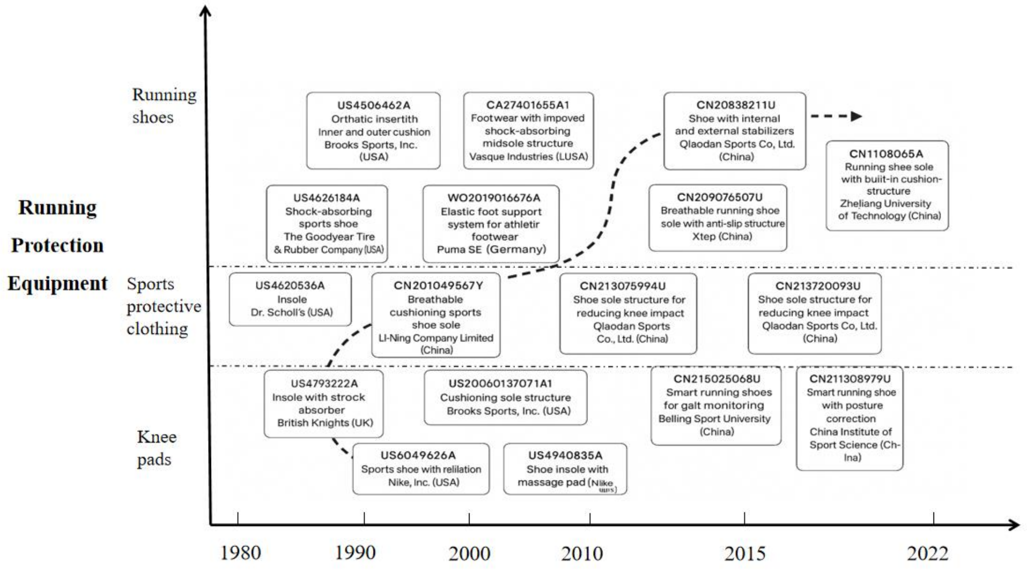

Figure 5 Technical Evolution of Running Protection Equipment

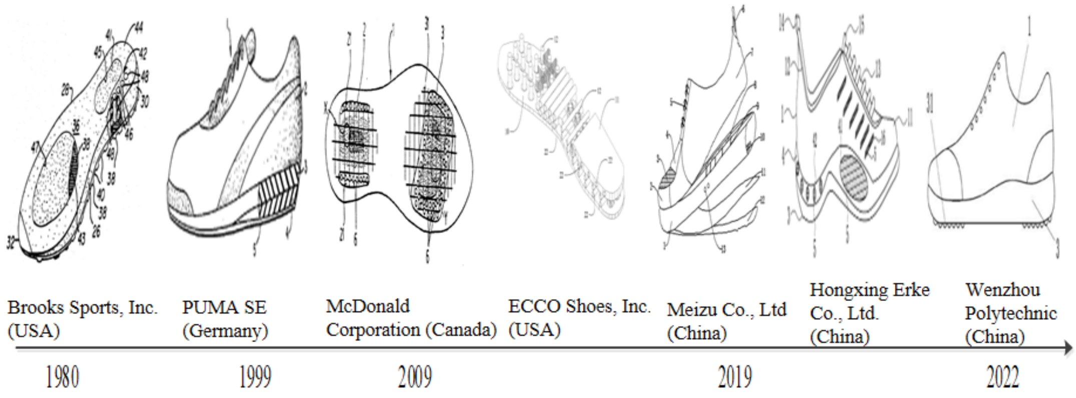

Figure 6 Technical Evolution of Running Shoes

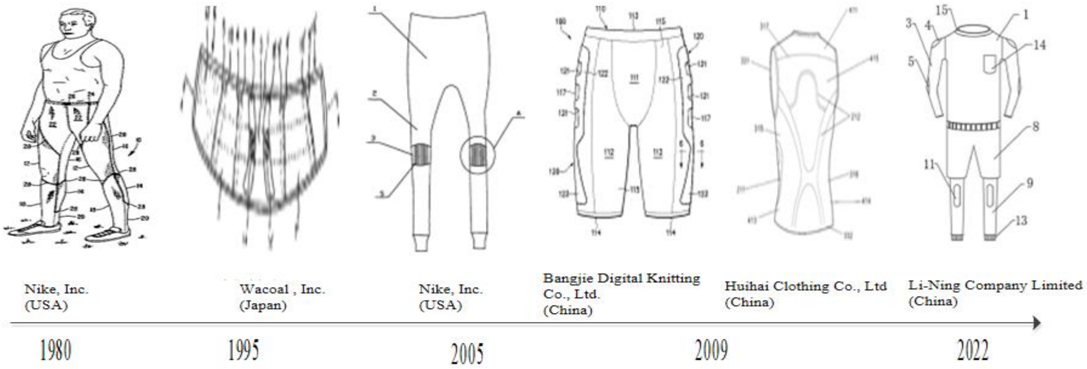

Figure 7 Technical Evolution of Sports Protective Clothing

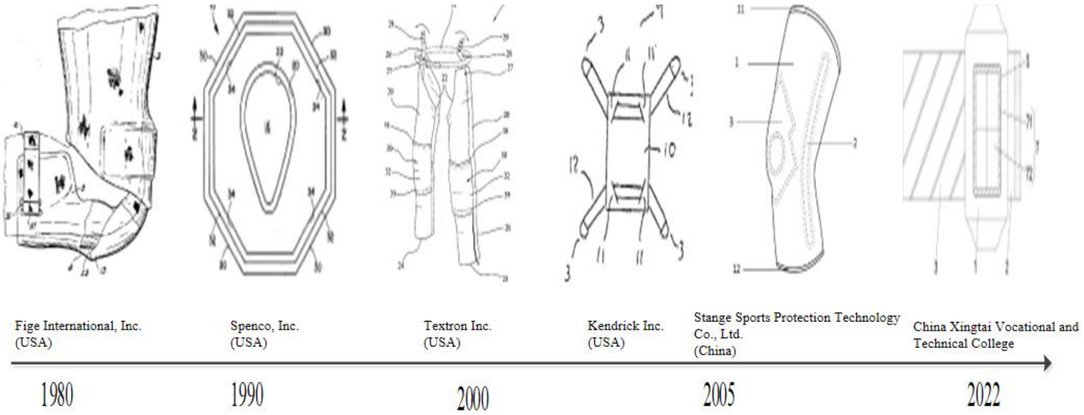

Figure 8 Evolution Diagram of Knee Support Technology
