# Supplementary material for: Research on the development trend of global running protection equipment technology from the perspective of patent measurement
Source: PLoS One. 2026 Jan 5;21(1):e0336148. doi: 10.1371/journal.pone.0336148 (PMC12768273; doi:10.1371/journal.pone.0336148)
Supplement: Attachment — Submitted filename: Response Letter.docx [file pone.0336148.s004.docx]

Dear Editors and Reviewers,

Thank you very much for handling our paper “PONE-D-25-26987” entitled “Research on the development trend of global running protection equipment technology from the perspective of patent measurement”. We are very grateful for your work and highly appreciate the constructive comments and suggestions made by the coordinating editors and reviewers. Based on the comments and suggestions, we carefully revised the manuscript. The quality of the manuscript has been greatly improved. Our point-by-point responses to comments from the reviewers are also uploaded.

Please let us know if additional information is needed.

Thank you very much for handling this manuscript.

Sincerely yours,

We highly appreciate the coordinating reviewers and editors for their valuable comments and suggestions. To our best, we managed to revise the manuscript point by point according to the comments right below this part. In the responses, we used different font style to facilitate reading as following.

- **Contents with underline are the original comments of the coordinating editors and reviewers.**
- Contents in normal Times New Roman are our response to the reviewers.

**Point by Point** **response to the coordinating reviewers and editors**

**Reviewer#1…………………………………………………………………….....…1-2**

**Reviewer#2………………………………………………………………………........3**

**Reviewer#3…………………………………………………………………….....…4-9**

**Reviewer#4……………………………………………………………………….10-11**

**Reviewer#5………………………………………………………………….....…12-17**

# Reviewer #1

## I commend the authors on the production of a very detailed and well researched paper. As a runner myself, I found the technical details fascinating and informative. However, as an academic with a primary interest in innovation and entrepreneurship, I felt that the details provided on the specifics of the innovation of individual items too detailed. The paper is very long and if I had been less interested in the innovation around these running technologies, I would have switched off halfway through the paper. I think you need to decide on who your target groups is specifically and perhaps edit the paper accordingly. The issues that are relevant, I feel are. The diffusion of innovation and the development trajectories (stages) of the market for these products. Linking this to the patent searches and the global development of running products is relevant and interesting. The stages of innovation of the products are also interesting, but too detailed. The point could have been made without such detailed accounts of the stages of innovation (although it did help me to understand why running shoes are so expensive). The protection of IP in China and the evolution of the process of IP protection was interesting and relevant. The paper makes the case for investment in this sector, which appears to be the main point of the paper. This brings me to the question of the purpose of the paper. If it is to inform ‘academics’ (researchers and student) in the process of innovation and patent development in China, using global running protection as a case in point, I feel its far too long. If it’s a pitch for development funding, then it makes its case well. The paper aims to provide a comprehensive analysis of global trends, but for what purpose? I feel this needs to be clearer, this is why I have suggestion minor revisions. The paper is well written, and I wish you every success with your publication of this work.

Our Response: We thank the reviewer for these thoughtful and constructive comments. We carefully considered the points raised and have revised the manuscript accordingly to ensure clarity, conciseness, and alignment of purpose.

①Simplification of innovation stages.

In the revised *Analysis of global patent application trends* section, we streamlined the discussion of innovation diffusion stages. The three phases (nascent stage 1898–1970, formative stage 1971–1999, and rapid advancement stage post-2000) are still clearly identified, but the descriptions have been significantly condensed. For example, instead of providing lengthy accounts of specific marathons or detailed lists of material innovations, we now emphasize patent counts and general technological directions. This change retains analytical rigor while avoiding unnecessary detail.

②Clarification of temporal coverage.

In the Data Sources and Search Strategy section, we now clarify that patents from 1898–1969 are included only as background information and not used for core analyses. The main analyses (e.g., technology evolution, quality evaluation) focus on 1970–2023, when patenting activity became more substantial. This adjustment ensures that early-stage data are properly contextualized without overburdening the discussion with sparse or anecdotal details.

③Clarification of research purpose.

Both the abstract and the conclusion have been revised to more clearly state the study’s purpose. Rather than implying theoretical model-building, the manuscript now emphasizes that it provides an evidence-based framework for understanding the global dynamics of running protective equipment technologies, with direct implications for patent management, industrial upgrading, and policy design. This resolves the ambiguity noted by the reviewer regarding the primary audience and objectives of the paper.

④Refinement of descriptive sections.

In response to the reviewer’s concern about promotional or overly descriptive passages, we have streamlined case descriptions involving companies such as Nike, Li Ning, and Waco. These are now presented solely as illustrative examples of patenting activity, accompanied by clarifying statements to ensure they are understood as empirical cases rather than endorsements. Figure captions have also been revised to focus on data sources and analytical insights instead of product-level details.

We believe that these revisions collectively address the reviewer’s concerns by simplifying overly detailed sections, clarifying the purpose of the paper, and ensuring that all analyses remain tightly linked to empirical results. We are grateful for the reviewer’s feedback, which has helped us to improve both the focus and the clarity of the manuscript.

# Reviewer #2

## This manuscript presents a thorough and well-structured analysis of global innovation trends in running protective equipment using patent data. The segmentation into three distinct phases of technological development (nascent, accumulation, and rapid growth) is supported by strong data and narrative coherence. The use of IPC classification and the TOPSIS model enhances the analytical depth.Strengths: The study provides a replicable and practical framework for countries seeking to evaluate or build their own innovation ecosystem in the sports equipment sector. It highlights China's growing role and offers policy suggestions, which could benefit both policymakers and industrial stakeholders. The structure, language, and flow of the manuscript are clear and logical. Suggested Minor Revision: **While the manuscript includes an excellent breakdown of valid vs. invalid patents (Figure 4), a brief reflection on how the high invalidation rate (over 50%) may affect the interpretation of total patent volume as an innovation indicator would strengthen the discussion. Specifically, clarifying whether trend charts and conclusions account for patent validity, citations, or other quality filters would enhance robustness. I recommend this paper for publication after minor revision.**

*Our Response*: We sincerely thank the reviewer for highlighting this important point. In response, we have revised the section following Figure 4 to explicitly acknowledge the limitations of interpreting total patent counts in light of the high invalidation rate. We now clarify that trend charts (Figures 1–3) and structural analyses (Tables 1–2) are based on overall filings, which primarily serve to capture the intensity and diffusion of inventive activity. To address the reviewer’s concern and ensure robustness, the subsequent analyses complement volume-based measures with quality-oriented indicators—including forward citation frequency, patent family size, number of claims, and survival period—as well as the TOPSIS-based identification of core patents. This integrated approach balances scale with quality, enabling a more rigorous and nuanced interpretation of innovation dynamics in the field of running protective equipment.

We also added a brief explanatory note to indicate that patents which later become invalid still provide meaningful evidence of inventive activity at the time of filing. This clarification is intended to strengthen the interpretation of our data, without diminishing the reviewer’s valid observation regarding the importance of validity-adjusted measures.

We believe these additions enhance the robustness and transparency of the manuscript, and we thank the reviewer again for this valuable suggestion.

# Reviewer #3

**This manuscript addresses the technological evolution of running protective equipment based on global patent data collected from the Patsnap database. The topic is timely and relevant given the increasing interest in sports injury prevention and sportswear innovation. However, there are multiple critical issues that need to be addressed before the manuscript can be considered for publication.**

## 1. Data Collection Transparency and Reproducibility: The manuscript lacks detailed information about how the patent data were collected. There is no disclosure of search queries, IPC codes, time filters, or keywords used in Patsnap. This makes the research irreproducible and undermines the validity of all quantitative results.

*Our Response:* We sincerely thank the reviewer for this valuable comment highlighting the importance of transparency and reproducibility in patent data collection. In the revised manuscript, we have substantially expanded the *Data Sources and Search Strategy* section to provide a detailed description of the data retrieval process. Specifically, we now clearly explain:

①Database and time frame: All patent data were collected from the Patsnap Global Patent Database, with the search finalized on December 31, 2023. The starting year of 1898 was chosen because manual inspection confirmed the earliest relevant patent dates back to that year, while the endpoint of 2023 was selected to account for the typical 18-month delay between patent application and publication, ensuring dataset completeness.

②Search strategy: A combined approach using keywords (applied to titles, abstracts, and claims) and IPC classifications was employed. The keywords included terms such as “protective running gear,” “running shoes,” “knee pad,” “waist support,” “sports protective clothing,” and “compression wear,” with Boolean operators and wildcards applied. The IPC whitelist covered footwear (A43B), protective clothing (A41D), and sporting equipment (A63B), together with relevant subclasses (e.g., A43B5/06, A43B13/18, A41D13/06, A63B71/12).

③Exclusion criteria and manual screening: Patents irrelevant to running protective equipment, such as industrial safety boots, ski boots, mountaineering boots, or medical orthoses, were excluded. In addition, we explicitly state that manual screening was conducted by reviewing abstracts and claims to further ensure the accuracy and relevance of the data set.

④Data cleaning: The data set was refined through INPADOC family aggregation, applicant name standardization and duplicate removal.

To maintain readability in the main text, only the key elements of the search and cleaning process are included in the Methods section. The full search queries, IPC class lists, exclusion criteria, and detailed cleaning procedures are provided in Appendices A–D for complete transparency and reproducibility.

## 2. Assumption of Patent Volume as a Proxy for Innovation: The authors repeatedly equate the number of patents with technological advancement without addressing the qualitative aspects of innovation. Defensive patents, duplicate filings, and low-quality submissions are not filtered or discussed.

*Our response*: We thank the reviewer for raising this important concern. We fully agree that patent counts alone cannot be equated with innovation, as they may be affected by defensive filings, duplicate family members, or low-quality submissions. In the original submission, this methodological clarification was not sufficiently emphasized.

In the revised manuscript, we have strengthened the *Data Sources and Search Strategy* section to explicitly acknowledge these limitations and to describe the safeguards applied in our study. We note that INPADOC family aggregation, duplicate removal, and manual screening of abstracts and claims were implemented to minimize the inclusion of duplicate filings, irrelevant applications, or purely defensive patents. Most importantly, we clarified that patent counts in this study are used only as a proxy for the intensity of inventive activity, while qualitative dimensions of innovation are addressed through complementary indicators.

Specifically, as elaborated in the sections Analysis of patent technology evolution and Core patent screening, our framework incorporates forward citation frequency (to capture technological impact and knowledge diffusion), patent family size (as a measure of international scope and strategic value), number of claims (as a proxy for technical breadth), and legal status such as survival period (to reflect durability). These indicators were combined through a weighted evaluation framework (TOPSIS) to identify core patents, ensuring that the analysis accounts for both the scale and the significance of technological innovation.

We believe these revisions make it clear that our methodology goes beyond patent volume and provides a more rigorous, balanced, and multi-dimensional assessment of innovation in the field of running protective equipment.

## 3. Lack of Methodological Rigor in Technology Evolution Analysis: The section on technological evolution (Figures 6–8) presents developmental trajectories without describing the analytical method used to derive them. There is no mention of citation networks, keyword co-occurrence, or other commonly used tools in patent mapping.

*Our response*: We thank the reviewer for raising this important point. In the revised manuscript, we have strengthened the methodological description to ensure that the procedures leading to Figures 6–8 are transparent and rigorous. Specifically, we clarified that the technology evolution analysis is based on a multi-step approach:

①A keyword–IPC dual filtering strategy was first applied to ensure both semantic relevance and technical specificity.

②A weighted TOPSIS evaluation was then used to identify core patents, incorporating forward citation frequency, patent family size, number of claims, and survival period.

③To enhance interpretive accuracy, we extended the manual review process beyond dataset cleaning to include the TOPSIS-identified core patents. Two coders with expertise in patent analysis and sports equipment technology examined abstracts, claims, and legal status, resolving discrepancies through discussion.

④Developmental trajectories were reconstructed from these validated core patents and synthesized into the maps presented in Figures 6–8.

We also modified the figure notes for Figures 5–8 to make clear that they are based on core patents identified via TOPSIS and validated through manual review.

These additions ensure that the methodological steps underpinning the technology evolution analysis are explicit, replicable, and fully consistent with the results presented.

## 4. Inconsistent Use of Classifications: IPC classifications are presented early on but are not consistently used to explain the technology evolution or policy suggestions. The analysis disconnects the quantitative classification data from the qualitative discussion in later sections.

*Our response*: We sincerely appreciate the reviewer’s insightful comment regarding the inconsistent use of IPC classifications across different sections of the manuscript. In response, we have carefully revised the paper to ensure that IPC classifications function as a consistent analytical thread connecting the quantitative and qualitative analyses, the policy recommendations, and the concluding remarks.

①The technology evolution analysis (Figures 5–8) has been substantially revised to explicitly align technological trajectories with IPC subclasses. For instance, running shoe innovations are now mapped to A43B subclasses such as A43B13/18 (stabilization plates), A43B13/12 (compressible midsoles), and A43B23/02 (structural reinforcements). Sports protective apparel is linked to A41D subclasses such as A41D31/02 (thermal regulation), A41D1/02 (abrasion resistance), and A41D13/05–06 (waist and knee protectors). Knee pad developments are anchored in A63B71/12 (protective devices for the body or legs), with subcategories corresponding to abrasion resistance, cushioning optimization, and impact protection. These revisions ensure that the qualitative discussion of material, structural, and functional innovations is directly grounded in the IPC classification framework presented earlier in the methodology.

②The policy recommendations section has been refined to incorporate IPC-based evidence in a systematic manner. For example, we emphasize that targeted government support should prioritize Chinese enterprises active in subclasses such as A43B5/06 (special-function footwear) and A41D13/06 (protective apparel for knees and feet), where our analysis identified both rapid growth and fragile competitiveness. Similarly, recommendations on collaborative patenting and commercialization are explicitly tied to the dominant IPC domains (A43B, A41D, and A63B), ensuring that strategic guidance is clearly rooted in the classification data used throughout the study.

③The conclusion has been rewritten to reinforce this consistency by summarizing technological trajectories explicitly through IPC categories. The concluding discussion highlights how A43B (footwear), A41D (protective clothing), and A63B (sporting equipment) provide a stable framework for interpreting long-term trends, while the identification of hotspots such as A43B13/18, A43B5/06, and A41D13/06 links the findings to concrete policy and industry implications.

Together, these revisions ensure that IPC classifications are not only introduced in the early methodological sections but are consistently applied in the analysis of technological evolution, the formulation of policy recommendations, and the articulation of conclusions. We believe this alignment strengthens the methodological rigor of the paper and enhances the interpretability and policy relevance of our findings.

## 5. Economic and Strategic Implications Lack Supporting Evidence: Policy and industry recommendations are made based on broad assumptions rather than evidence directly derived from the data. For example, the argument that low patent validity rates indicate a lack of commercialization is not supported by further economic data, case studies, or firm-level analysis.

*Our response*: We sincerely thank the reviewer for this valuable comment. We fully agree that the initial version of our manuscript presented policy and industry recommendations that were described too generally and insufficiently linked to our empirical findings. In response, we have substantially revised the “Suggestions and Policy Recommendations” section to ensure that each recommendation is directly grounded in the analyses of patent data presented in Sections Analysis of basic patent information and Analysis of patent technology evolution. The key improvements are as follows:

①Linking IP governance recommendations to legal status analysis

*****In the revised text, recommendations regarding strengthening IP governance and patent quality are explicitly anchored to our legal status results, which showed that only 34.47% of patents remain valid and that average maintenance periods are short.

*****Instead of broadly assuming a lack of commercialization, we now argue that premature lapses primarily reflect weak maintenance incentives and quality gaps. We emphasize that institutional reforms should be targeted at extending the survival of high-quality patents identified in our TOPSIS screening, thereby aligning governance with empirical evidence.

②Grounding enterprise-level recommendations in applicant and IPC distributions

*****Our revision highlights that while China accounts for the largest number of filings, patents with higher forward citations and longer maintenance are concentrated in firms from advanced economies (United States, Germany, Japan).

*****This finding justifies policy measures to foster domestic leading enterprises, with explicit focus on IPC subclasses showing both technological momentum and fragility (e.g., A43B5/06 running shoes, A41D13/06 knee protectors). In this way, government support is directly tied to the technological hotspots identified in our data set.

③Aligning strategic patent management with technological evolution trends

*****Based on Figures 6–8, which illustrate the shift from structural stabilization to advanced materials and intelligent features, we revised the recommendations to emphasize that Chinese firms should strategically enter emerging domains rather than focusing solely on traditional technologies.

*****We further integrate our TOPSIS-based quality indicators, suggesting that firms prioritize patents with strong forward citations, larger families, and longer survival periods to ensure resilient and commercially relevant portfolios.

④Revising the commercialization discussion with evidence-based proxies

*****We acknowledge that validity rates alone cannot fully capture commercialization. Therefore, in the revised section we add that commercialization potential should be inferred from legal-event proxies available in patent data, such as assignments, licensing, and pledges.

*****By integrating these indicators with our TOPSIS results, we provide a more rigorous and evidence-driven basis for recommending “use-oriented” patent strategies. We also connect this recommendation to the observed thin and fragmented co-assignee networks, highlighting the need for stronger collaboration between industry, academia, and research institutions.

Overall, these revisions directly address the reviewer’s concern by embedding our recommendations into the empirical findings of the study. Rather than being based on broad assumptions, the revised recommendations are explicitly supported by data-driven evidence from patent validity rates, IPC trends, technological evolution patterns, and quality-based indicators. We believe this significantly enhances the rigor and credibility of the policy and industry implications presented in the manuscript.

## 6. Minor Issues: Some sections are overly descriptive (e.g., product features from companies like Nike, Li Ning, or Waco), giving the impression of promotional content rather than neutral academic analysis.

*Our response*: We thank the reviewer for this constructive observation. We fully agree that certain passages in the original manuscript contained descriptions of company products that may have inadvertently created the impression of promotional language.

In the revised manuscript, we carefully reviewed all sections involving company-related content (e.g., Nike, Li Ning, Waco, Brooks, Puma, Hongxing Erke, etc.). Overly descriptive expressions such as “remarkable vitality,” “undisputed leadership,” “absolutely dominant position,” or detailed product specifications have been either removed or rewritten in a more neutral, academic style. Specifically, we:

①Reframed product-oriented descriptions into technology- and patent-oriented analyses. For example, instead of describing Nike’s product features, we now state that “patents attributed to Nike focus on mesh-based fabrics and thermostatic components aimed at improving thermal regulation.”

②Consolidated scattered product details into broader technical categories (e.g., cushioning systems, breathable materials, structural reinforcements), thus shifting the emphasis from individual products to technological trends.

③Added clarifying statements that these examples serve only as illustrative cases of patenting activity and not as endorsements of specific companies or products.

## 7. Figures lack explanatory captions and do not cite the specific patents or datasets they are based on.

*Our response*: We sincerely thank the reviewer for this helpful observation. We agree that the figure captions in the original submission were too concise and did not provide sufficient explanatory detail or references to the underlying data and representative patents.

In the revised manuscript, we have comprehensively updated all figure captions to improve their clarity and informativeness. Specifically, each figure caption now:

①Specifies the data source and retrieval date (Patsnap Global Patent Database, January 2025).

②Defines the scope of data collection, including the relevant IPC classes (A43B, A41D, A63B) and, where applicable, the subclasses used.

③Describes the methodological treatment, such as INPADOC family aggregation, duplicate removal, applicant name harmonization, and manual screening.

④Includes representative patents in figures illustrating technological evolution (e.g., running shoes, protective clothing, and knee braces).

⑤Provides a concise explanation of the main findings that the figure illustrates (e.g., temporal trends, country distributions, or pathways of technological development).

We believe these revisions significantly enhance the interpretability, transparency, and reproducibility of our results.

## 8. The time span from 1898 to 2023 is unnecessarily broad; data before the 1970s are too sparse to support meaningful analysis.

*Our response*: We thank the reviewer for this valuable comment. We agree that the number of patents prior to the 1970s is extremely limited and insufficient to support systematic statistical analysis. In the original version, our rationale for selecting 1898 as the starting point was not clearly explained.

In the revised manuscript, we clarify that the year 1898 was chosen because manual inspection confirmed it as the earliest filing date of a patent related to running protective equipment. This choice ensures historical completeness and provides context on the field’s earliest technological explorations. However, we also acknowledge that data before the 1970s are sparse. To address this limitation, we explicitly state that patents from 1898–1969 are presented only as background information to illustrate the emergence and early development of protective technologies, while the core analyses—including technology evolution trends, core patent screening, and quality-oriented evaluations—are based primarily on the 1970–2023 period, when patenting activity expanded significantly. Furthermore, sparse early data are flagged and aggregated in the figures to prevent them from distorting the overall trends.

# Reviewer #4

## The article seems a software analyzed than author, seek to have excel data verification to validate. The technology details are generic not citing proper patent documents, Global analysis is skeptical.. some reference of revenue generated by IP has to be explained how calculated for all these patents.. suggestion has to be trimmed down to exactly talk about subject matter technology and suggestion.. generic suggestion policy reforms is fine but talking protection equipment technology is essential and the final conclusion are very generic which needs to be specific to subject matter discussed

*Our response*: We are sincerely grateful for this detailed and constructive comment. It has helped us identify weaknesses in the original manuscript and guided us to make substantial revisions. We have worked carefully on multiple levels—methods, evidence, analysis, policy, and conclusions—to address each of the concerns raised.

①Regarding the impression that the analysis appeared software-driven rather than author-led, we greatly expanded the *Data Sources and Search Strategy* and *Core Patent Screening* sections. The revised version now specifies the use of a keyword–IPC dual filtering strategy applied to titles, abstracts, and claims, followed by INPADOC family aggregation, duplicate removal, and applicant name harmonization. We emphasized that these procedures follow internationally recognized methodological standards, citing the OECD Patent Statistics Manual and Grupp & Schmoch (1999). To strengthen interpretive rigor, we adopted a multi-criteria TOPSIS framework that integrates citation counts, family size, claims, and survival period. Most importantly, we complemented this with expert involvement: subjective weights were derived from specialists in intellectual property, sports equipment technology, and patent analysis, and two independent coders manually reviewed the shortlisted patents. These additions make clear that the results are author-driven, transparent, and reproducible, rather than the product of black-box software.

②In response to the comment that the technology discussion was too generic and not linked to patents, we rewrote the *Technology Evolution Analysis* and revised Figures 5–8. The current version explicitly anchors innovations to IPC subclasses and representative patents. For example, running shoe technologies are mapped to A43B13/18 (stabilization plates, Brooks), A43B13/12 (compressible midsoles, Puma), and A43B23/02 (reinforcements, Erke). Protective apparel developments are linked to A41D31/02 (thermal regulation, Nike) and A41D13/06 (knee and waist protectors, Waco and Chinese patents). Knee pad advances are connected to A63B71/12 (protective devices, Fige International, Textron, and others). By aligning each trajectory with both IPC codes and illustrative patents, we sought to transform what was previously a generic description into a verifiable, evidence-based account.

③On the reviewer’s skepticism regarding global analysis, we clarified in the *Data Sources and Search Strategy* and *Analysis of global patent application trends* section that although the dataset covers 1898–2023, patents before 1970 are extremely sparse and cannot support statistical inference. These early patents are retained only as descriptive background to illustrate initial experimentation, while all substantive analyses are restricted to 1970–2023, when patent activity expanded significantly. Early data are separately flagged in figures to prevent distortion. This ensures that the global analysis is robust, empirically cautious, and not overstated.

④With respect to intellectual property revenue, we revised the *Legal Status Analysis* to make our approach more rigorous and transparent. We do not attempt to calculate revenue directly; instead, we use validity rates and survival periods as proxies for commercialization potential. Our results show that only 34.47% of patents remain valid, while more than half have lapsed—23.05% due to non-payment of fees and 10.98% due to withdrawal. We also highlight legal events such as assignments, licenses, and pledges as additional indicators of commercialization activity. These revisions provide a scientifically defensible and widely accepted way to interpret economic viability without relying on speculative monetary estimates.

⑤The reviewer suggested that our policy recommendations were too general. We have therefore restructured and trimmed this section to align closely with the technologies studied. The revised recommendations explicitly reference IPC hotspots such as A43B5/06 (special-function footwear) and A41D13/06 (knee/foot protectors). They also build directly on our empirical findings, linking government support and procurement priorities to applicant distributions, forward citation performance, and the presence of legal events. While we still acknowledge systemic IP governance issues, the emphasis is now firmly placed on running shoes, protective apparel, and knee pads, ensuring that our suggestions are specific to the subject matter.

⑥To respond to the concern that the conclusion was overly generic, we completely rewrote this section to make it specific to the study. The revised conclusion synthesizes findings across the three IPC domains of footwear (A43B), protective apparel (A41D), and knee pads (A63B). It highlights concrete technological hotspots such as stabilization plates (A43B13/18), cushioning systems (A43B13/12), thermal regulation (A41D31/02), and shock-absorbing devices (A63B71/12). It also integrates legal status evidence, noting that only one-third of patents remain valid, to underline sustainability challenges. By embedding IPC-based evidence, quality indicators, and applicant profiles, the conclusion is now more precise and directly aligned with the empirical analysis.

In summary, we have worked extensively to ensure that the revised manuscript addresses each of the reviewer’s concerns. We reinforced methodological rigor to show the analysis is author-led, integrated IPC and patent references to eliminate generic descriptions, contextualized the global analysis to ensure robustness, replaced speculative revenue with legal status evidence, focused recommendations on subject-specific technologies, and rewrote the conclusion to be concrete and aligned with the data. We are deeply grateful for this feedback, which has pushed us to strengthen the manuscript in multiple dimensions. While we recognize there is always room for further refinement, we believe the revisions represent a significant step forward in clarity, rigor, and relevance.

# Reviewer #5

## (1) This imbalance poses significant challenges to commercialization and limits the economic viability of innovations. Current R&D efforts are primarily focused on running shoes, sportswear, and knee pads. Focus on your research key results.

*Our response*: We thank the reviewer for this insightful comment. We agree that in the original version, the discussion of “imbalance” and its implications for commercialization was presented in general terms without sufficient grounding in our empirical findings. In the revised abstract, we have addressed this issue by explicitly linking these statements to the results of our patent analyses.

Specifically, we now state that only 34.47% of patents in the data set remain valid, with more than half lapsing within a relatively short period. This result, derived from our analysis of patent validity, demonstrates weak incentives for long-term maintenance and provides concrete evidence of structural weaknesses in sustaining innovation. Furthermore, based on the results of our technological evolution analysis, we emphasize that innovation activity is highly concentrated in three domains—running shoes, sportswear, and knee pads—while diversification into emerging intelligent and material-enhanced technologies remains limited.

By grounding the description of “imbalance” in these findings, the revised abstract now highlights that the commercialization challenges and constrained economic viability observed in this sector are directly linked to the low durability of patents and the narrow concentration of technological domains identified in our data set. We believe this revision successfully addresses the reviewer’s concern by ensuring that the abstract is tightly focused on our key research results.

## (2) This analysis aims to provide theoretical support for decision-making in technology R&D and patent strategies for China’s running protective equipment industry. Whereas the study does not provide theoretical support, therefore authors must align findings and aims of this research.

*Our response*: We thank the reviewer for pointing out this inconsistency between the stated aims and the actual contributions of our study. We fully agree that the manuscript should not overstate the provision of “theoretical support,” as our research is primarily grounded in empirical patent data analysis rather than theoretical model construction. To address this concern, we have undertaken the following revisions:

①Reframing the research aim and contribution.

*****The phrase “*provide theoretical support*” has been replaced with: “*This analysis provides an evidence-based foundation for decision-making in technology R&D and patent strategies in China’s running protective equipment industry, offering empirical insights into patent quality, technological trajectories, and commercialization potential.*” This revision aligns the aim of the study more closely with its empirical nature and contributions.

②Strengthening the link between findings and implications.

*****As reflected in our revised abstract and policy recommendations, the imbalance in patent validity (only 34.47% remain active), the concentration of innovation domains (running shoes, sportswear, knee pads), and the identification of high-quality patents through TOPSIS analysis are now explicitly connected to the strategic implications for R&D, patent management, and commercialization.

*****By grounding recommendations in these results, the manuscript now demonstrates that its practical significance derives directly from empirical evidence rather than from abstract theorization.

③Ensuring consistency across sections.

*****Corresponding adjustments were made in the conclusion and suggestion sections to ensure that the language used to describe the study’s contribution is consistent with its findings, thereby avoiding overstated claims and reinforcing academic rigor.

We believe these revisions have resolved the reviewer’s concern by ensuring that the aims, findings, and implications of the research are fully aligned and accurately presented.

## (3) In this study, the terms “Protective running gear” and “Running protective gear” were initially employed to conduct a search in the Patsnap global patent database. Logical ground is missing, could you please provide proper references and base, or is this methodology suitable for this study?

*Our response*: We thank the reviewer for this insightful comment. We recognize that in the original submission the rationale for our search terminology was not sufficiently elaborated. In the revised manuscript, we have expanded the *Data Sources and Search Strategy* section to provide a clear methodological justification and relevant references.

Specifically, we explain that the retrieval employed a combined keyword–classification approach, which is widely acknowledged as a robust methodology in patent analysis. The terms “protective running gear” and “running protective gear” were selected as core keywords because they directly correspond to the thematic scope of this study. To strengthen coverage and reduce retrieval bias, the keyword set was expanded to include semantically and technically related terms such as “running shoe” “sports footwear” “knee pad” “knee brace” “waist support” “sports protective clothing” and “compression wear”. These terms were searched across the title, abstract, and claims fields with Boolean operators and wildcards applied.

To further improve precision, the keyword searches were combined with International Patent Classification (IPC) codes related to footwear, protective clothing, and sporting equipment (A43B, A41D, A63B, and their relevant subclasses). This dual-filtering strategy (keywords+IPC classes) aligns with international best practices in patent statistics and has been explicitly recommended in authoritative manuals such as Patents as Statistical Indicators of Science and Technology (OPS, 2009) and Indicators of the Internationalisation of Science and Technology (OPS, 2009). Moreover, it is consistent with prior empirical research, such as Grupp and Schmoch (1999), which demonstrated the importance of integrating semantic search terms with classification systems to ensure precision in patent-based analyses.

In addition, we describe that irrelevant patents (e.g., industrial safety boots, mountaineering footwear, ski boots, and medical orthoses) were systematically excluded, while the final data set was validated through INPADOC family aggregation, applicant name harmonization, and manual inspection of abstracts and claims.

These revisions establish that the chosen search methodology is both methodologically sound and well-grounded in the existing literature, thereby ensuring transparency, reproducibility, and suitability for the objectives of this study.

## (4) It is recommended to add a proper section of methodology.

*Our response*: We sincerely appreciate the reviewer’s suggestion regarding the methodology section. In the initial submission, details of the data collection and processing strategy were not fully elaborated, which may have limited the transparency and reproducibility of the study. To address this concern, we have substantially revised and expanded the section now titled “Data Sources and Search Strategy” to ensure methodological rigor. The revised section now includes the following enhancements:

①Temporal coverage and justification.

*****We explicitly state that the data set covers 1898–2023, with clear rationale for choosing the starting and ending years.

*****To address potential concerns about sparse early data, we clarify that patents from 1898–1969 are included only as historical background, while the core analyses are restricted to 1970–2023. Early data are flagged and aggregated in figures to avoid distortion.

②Search strategy and filtering procedures.

*****We describe in detail the dual-filtering strategy combining keywords (e.g., “protective running gear” “running shoes” “knee pad” etc.) and IPC classifications (e.g., A43B, A41D, A63B).

*****High-frequency subclasses such as A43B5/06, A43B13/18, A41D13/06, and A63B71/12 were validated through iterative testing and manual verification.

*****This approach is explicitly linked to established guidelines (OECD Patent Statistics Manual) and prior empirical studies to demonstrate methodological reliability.

③Data cleaning and quality control.

*****We add a systematic description of INPADOC family aggregation, duplicate removal, applicant name harmonization, and manual review of abstracts and claims.

*****Exclusion criteria are detailed (e.g., industrial safety boots, ski boots, medical orthoses) to ensure dataset relevance.

④Addressing the “patent volume as proxy for innovation” limitation.

*****Recognizing the reviewer’s concern, we note that patent counts alone can be misleading.

*****We therefore incorporate quality-oriented indicators such as forward citations, family size, number of claims, and survival period to provide a more comprehensive and balanced measurement of innovation.

Through these revisions, the methodology section now presents a transparent, reproducible, and scientifically rigorous framework for patent data analysis. We believe this addition fully addresses the reviewer’s comment and strengthens the methodological contribution of the study.

## (5) From the perspective of legal status, a total of 1,519 patents in the field of running protective equipment are currently authorized and valid, accounting for 34.47% of all filings. Add citations for such numeric values in the whole manuscript.

*Our response*: We greatly appreciate the reviewer’s careful reading and constructive suggestion. We acknowledge that the numeric values reported in the original manuscript lacked explicit source citation, which may affect the transparency and credibility of our results. In the revision, we have addressed this issue in the following ways:

①Explicit indication of data source in the main text. All numeric values related to patent counts, percentages, and legal status have been revised to specify their source. For example, the sentence has been revised to: “From the perspective of legal status, a total of 1519 patents in the field of running protective equipment are currently authorized and valid, accounting for 34.47% of all filings, based on the authors’ statistical analysis using the Patsnap Global Patent Database (retrieved Jan 2025) [15].”

②Clarification in the methodology section. We have added a dedicated statement in the “Data Sources and Search Strategy” section to clarify the database and retrieval process, as follows: “Patent data for this study were collected from the Patsnap Global Patent Database, and the retrieval was conducted in Jan 2025. The data were subsequently processed and summarized by the authors to derive descriptive statistics (e.g., counts, proportions, and legal status distribution).”

③Addition of a standardized reference. To ensure proper citation, we have added the following entry to the reference list: “Patsnap. Patsnap Global Patent Database [Internet]. Singapore: Patsnap Pte. Ltd.; 2025 [cited 2025 Jan 15]. Available from: https://analytics.zhihuiya.com/search/input/simple#/simple.”

Through these revisions, all numeric values in the manuscript are now accompanied by clear source attribution, and the methodological transparency of the study has been enhanced.

## (6) To ensure a scientifically rigorous assessment and accurately identify high-quality patent samples, the evaluation framework incorporates both subjective and objective factors. Whereas, subjective and objective factors must be stated here.

*Our response*: We sincerely thank the reviewer for this important suggestion. In the original version, we did not explicitly specify the subjective and objective factors included in the evaluation framework. We have now carefully revised the manuscript to address this issue.

In the modified section, we explicitly distinguish between subjective and objective factors. The subjective factors were derived from expert evaluations of the relative importance of the three dimensions—technological advancement, application prospects, and operational stability. To ensure the reliability and representativeness of these judgments, we invited three categories of experts: (1) university scholars specializing in intellectual property and innovation management; (2) technical professionals working in the sports equipment industry; and (3) patent analysts with more than two years of experience in patent evaluation. Their combined expertise provided theoretical, practical, and analytical perspectives, which compensate for the limitations of purely quantitative data. The objective factors are measurable indicators obtained from the patent database and include: (1) in the dimension of technological advancement, the number of cited patent documents, forward citation frequency, and the number of patents; (2) in the dimension of application prospects, the number of IPC classifications, the number of claims, and patent family size; and (3) in the dimension of operational stability, the number of pledges/transfers/licenses, the survival period, and the number of specification pages. The objective weights were calculated using the entropy method, and then integrated with the subjective weights to form the final combined weights, which were subsequently applied within the TOPSIS model to compute the comprehensive scores of patents. These revisions clarify the definition and role of subjective and objective factors, while also strengthening the transparency and scientific rigor of the methodological framework.

## (7) In the initial applications of knee pads, functional limitations were evident due to coarse surface friction treatments and inadequate fixation mechanisms, resulting in frequent slippage and displacement among runners. To address these issues, Fige International Company (United States) developed an innovative knee brace featuring a double-layer structure with a specialized pad arranged in an overlapping composite configuration. It is recommended to add the specification of the knee brace by explaining how it overcomes the stated issues.

*Our response*: We sincerely thank the reviewer for this valuable suggestion. In the original version, our description of the knee brace was limited to its general structure, without providing sufficient details on the technical specifications. We have carefully revised the manuscript to address this concern.

In the revised text, we have incorporated explicit specifications drawn from the patent description to explain how the device addresses the problems of slippage, displacement, and insufficient shock absorption observed in early knee pads. The revised section now highlights that the brace features a contoured concavity in the upper region that accommodates and laterally supports the patella, with the depth of the concavity restricted to no more than approximately 65% of the overall pad thickness, thereby ensuring both stability and mobility. The cushioning section is made of viscoelastic polyurethane foam with a storage modulus controlled within the range of 10–45 psi/cycle, which effectively absorbs vertical impacts while minimizing rebound forces. The contact surface is composed of a soft, breathable fabric to enhance comfort and prevent staining of the knee or clothing. The lower region is constructed from ethylene vinyl acetate (EVA), which maintains structural stability and provides reliable frictional resistance against the supporting surface without sticking.

These specifications clearly demonstrate how the patented brace design successfully overcomes the limitations of earlier products and substantively improve stability, comfort, and protective performance during vigorous exercise.
